# Subclassification of obesity for precision prediction of cardiometabolic diseases

**DOI:** 10.1038/s41591-024-03299-7

**Published:** 2024-10-24

**Authors:** Daniel E. Coral, Femke Smit, Ali Farzaneh, Alexander Gieswinkel, Juan Fernandez Tajes, Thomas Sparsø, Carl Delfin, Pierre Bauvin, Kan Wang, Marinella Temprosa, Diederik De Cock, Jordi Blanch, José Manuel Fernández-Real, Rafael Ramos, M. Kamran Ikram, Maria F. Gomez, Maryam Kavousi, Marina Panova-Noeva, Philipp S. Wild, Carla van der Kallen, Michiel Adriaens, Marleen van Greevenbroek, Ilja Arts, Carel Le Roux, Fariba Ahmadizar, Timothy M. Frayling, Giuseppe N. Giordano, Ewan R. Pearson, Paul W. Franks

**Affiliations:** 1 Genetic and Molecular Epidemiology Unit, Lund University Diabetes Centre, Department of Clinical Science, Lund University, Helsingborg, Sweden; 2 Maastricht Centre for Systems Biology (MaCSBio), Maastricht University, Maastricht, The Netherlands; 3 Department of Epidemiology, Erasmus MC University Medical Center, Rotterdam, The Netherlands; 4 Preventive Cardiology and Preventive Medicine, Center for Cardiology, University Medical Center of the Johannes Gutenberg-University Mainz, Mainz, Germany; 5 Department of Pharmacometrics, Novo Nordisk A/S, Søborg, Denmark; 6 Université de Lille, Inserm, CHU Lille, Institut Pasteur de Lille, U1190-EGID, Lille, France; 7 Biostatistics and Bioinformatics, Milken Institute School of Public Health, George Washington University, Rockville, MD USA; 8 Biostatistics and Medical Informatics Research Group, Department of Public Health, Vrije Universiteit Brussel, Brussels, Belgium; 9 Nutrition, Eumetabolism and Health Group, Institut d’Investigació Biomèdica de Girona (IDIBGI-CERCA), Girona, Spain; 10 Department of Medical Sciences, University of Girona, Girona, Spain; 11 CIBER Fisiopatología de la Obesidad y Nutrición (CIBEROBN), Instituto de Salud Carlos III, Madrid, Spain; 12 Department of Diabetes, Endocrinology and Nutrition, Dr. Josep Trueta University Hospital, Girona, Spain; 13 Departments of Epidemiology, Erasmus MC University Medical Center, Rotterdam, The Netherlands; 14 Diabetic Complications Unit, Lund University Diabetes Centre, Department of Clinical Science, Lund University, Malmö, Sweden; 15 Translational Medicine and Clinical Pharmacology, Boehringer Ingelheim Pharma GmbH & Co. KG, Ingelheim am Rhein, Germany; 16 Center for Thrombosis and Haemostasis, University Medical Center of the Johannes Gutenberg-University Mainz, Mainz, Germany; 17 DZHK (German Center for Cardiovascular Research), Partner Site Rhine-Main, Mainz, Germany; 18 Institute of Molecular Biology (IMB), Mainz, Germany; 19 School for Cardiovascular Diseases, Maastricht University, Maastricht, The Netherlands; 20 Diabetes Complications Research Centre, Conway Institute, University College Dublin, Dublin, Ireland; 21 Data Science and Biostatistics Department, Julius Global Health, University Medical Center Utrecht, Utrecht, The Netherlands; 22 Department of Epidemiology, Harvard T.H. Chan School of Public Health, Boston, MA USA; 23 Genetics of Complex Traits, College of Medicine and Health, University of Exeter, Exeter, UK; 24 Population Health and Genomics, University of Dundee, Dundee, UK

**Keywords:** Obesity, Diagnostic markers

## Abstract

Obesity and cardiometabolic disease often, but not always, coincide. Distinguishing subpopulations within which cardiometabolic risk diverges from the risk expected for a given body mass index (BMI) may facilitate precision prevention of cardiometabolic diseases. Accordingly, we performed unsupervised clustering in four European population-based cohorts (*N* ≈ 173,000). We detected five discordant profiles consisting of individuals with cardiometabolic biomarkers higher or lower than expected given their BMI, which generally increases disease risk, in total representing ~20% of the total population. Persons with discordant profiles differed from concordant individuals in prevalence and future risk of major adverse cardiovascular events (MACE) and type 2 diabetes. Subtle BMI-discordances in biomarkers affected disease risk. For instance, a 10% higher probability of having a discordant lipid profile was associated with a 5% higher risk of MACE (hazard ratio in women 1.05, 95% confidence interval 1.03, 1.06, *P* = 4.19 × 10^−10^; hazard ratio in men 1.05, 95% confidence interval 1.04, 1.06, *P* = 9.33 × 10^−14^). Multivariate prediction models for MACE and type 2 diabetes performed better when incorporating discordant profile information (likelihood ratio test *P* < 0.001). This enhancement represents an additional net benefit of 4−15 additional correct interventions and 37−135 additional unnecessary interventions correctly avoided for every 10,000 individuals tested.

## Abstract (web summary)

A precision medicine approach used unsupervised clustering to identify five distinct phenotypic profiles that can better predict risks of cardiometabolic disease compared with those ascertained based on the additive value of body mass index and other biomarkers, and validated these findings across four independent cohorts.

## Main

Obesity is steadily rising worldwide, with one in five of the world’s population, 1.5 billion people, projected to have obesity by 2030 (ref. ^1^), leading to higher risk of life-threatening conditions such as cardiovascular diseases (CVD) and type 2 diabetes (T2D)^2^. Along with the sheer numbers of those affected, prevention and care in obesity are further complicated by the complex and heterogeneous nature of these associations. This variation in comorbidities and phenotypes suggests that informative subclassification of obesity might facilitate precision medicine approaches for prevention and treatment.

BMI, the common metric used by epidemiologists, health professionals and others to characterize obesity, is easy to obtain and correlates well at a population level with gold-standard measures of adiposity^3^. However, BMI is insufficient for accurate classification of the disease of obesity at an individual level because people with similar BMIs often exhibit disparate health risks^4^. This is partially because BMI is an imperfect measure of excess adiposity that does not distinguish the proportion or distribution of fat mass and fat-free mass in the body^5^. Thus, clinicians use BMI for screening, although always in conjunction with other risk measures^6^.

There are established inconsistencies in the relationship between BMI and cardiometabolic disease^7,8^. Common signs of metabolic dysfunction such as insulin resistance and hypertension are absent in around 7% of individuals whose BMI is above the threshold for obesity^9^. Moreover, about 20% of individuals have multiple cardiometabolic risk factors despite being within the normal weight range of BMI^10^. Certain individuals are particularly sensitive to lifestyle exposures that impact BMI and CVD^11^. There are also diverse genetically determined obesity phenotypes, each conveying distinct metabolic signatures and varying levels of CVD risk^12,13^. Although these observations suggest the existence of subgroups at disproportionately higher or lower risk of CVD, they also underscore the challenges involved in accurately identifying individuals in these subgroups.

In this analysis, we used an ensemble of clustering techniques to decompose the general population into profiles that represent phenotypic ‘discordance’ deviating from the ‘concordant’ profile, which represents the linear relationship between clinical measures and BMI. Instead of categorically assigning individuals to specific profiles, each individual is assigned allocation probabilities for all profiles. Together these profiles present a more systematic evaluation of the various obesity-related cardiometabolic phenotypes than has previously been established. We explore the characteristics of these profiles and their potential clinical implications for cardiometabolic risk in four large independent cohorts across Europe.

## Results

### BMI–biomarker discordance

**Fig. 1:**
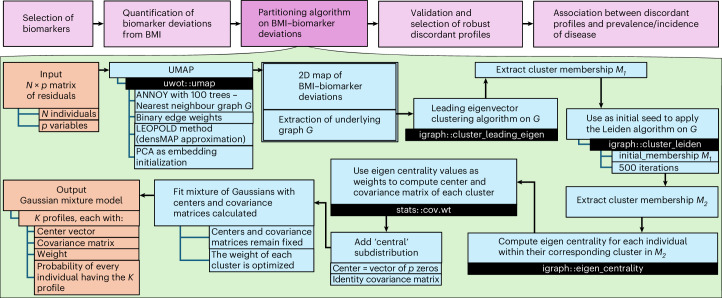
Study workflow. Flowchart depicting the overall steps in our analysis of BMI–biomarker discordance, with details about the ensemble of algorithms used to partition BMI–biomarker discordance into probabilistic profiles. PCA, principal components analysis.

An overview of the analysis pipeline is depicted in Fig. 1. We defined BMI–biomarker discordance as the residuals from the BMI-based predictions while adjusting for age and smoking status. To define this discordance, we used data at recruitment from the European ancestry subset of the UK Biobank (UKB) (*N* = 145,111) as our discovery dataset to identify BMI-discordant phenotypes. We did not use any BMI threshold for inclusion. We selected 10 BMI-related biomarkers routinely used in the clinical setting for risk stratification, each representing readouts from different biological processes known to be affected by obesity: fasting glucose (FG), representing glycemia; lipid fractions (high-density lipoprotein (HDL), low-density lipoprotein (LDL), triglycerides (TG)), representing lipid metabolism; systolic and diastolic blood pressures, representing hemodynamic function; serum creatinine, representing renal function; alanine transaminase (ALT), representing hepatic function; C-reactive protein (CRP), representing the inflammatory system; and waist-to-hip ratio, representing adipose distribution. We then conducted sex-specific analyses to quantify the BMI–biomarker relationships (Supplementary Fig. 1) and BMI-discordance for individual measurements. A significantly higher proportion of individuals displayed substantial discordance than the anticipated proportion under a normal distribution around BMI-based expectations (expected proportion = 5%, observed proportion = 10.3%; *P*_binomial_ < 0.001).

### Visualization and clustering of discordant profiles

We constructed a proximity network using biomarker deviations and visualized this network in two-dimensional (2D) projections using the uniform manifold approximation and projection (UMAP) method^14^. Individuals with substantial discordance appear to cluster within subgroups, a pattern absent in projections under a normal distribution (Supplementary Figs. 2 and 3). Linear dimensionality reductions (that is, principal component analysis) were unable to capture this discordance, likely because of the relatively low proportion of variance explained by the first two principal components (~35%) and the monotonicity of the cumulative variance explained (Supplementary Fig. 4).

To ascertain the subgroups observed in the UMAP projections, we deployed a soft-clustering algorithm on UMAP’s underlying proximity network. Briefly, this method converts partitions produced by a series of iterative graph-clustering techniques to a Gaussian mixture distribution. Individuals were thus assigned allocation probabilities to all subgroups rather than categorical allocations to a single subgroup. We used these allocation probabilities in all downstream analyses.

**Fig. 2:**
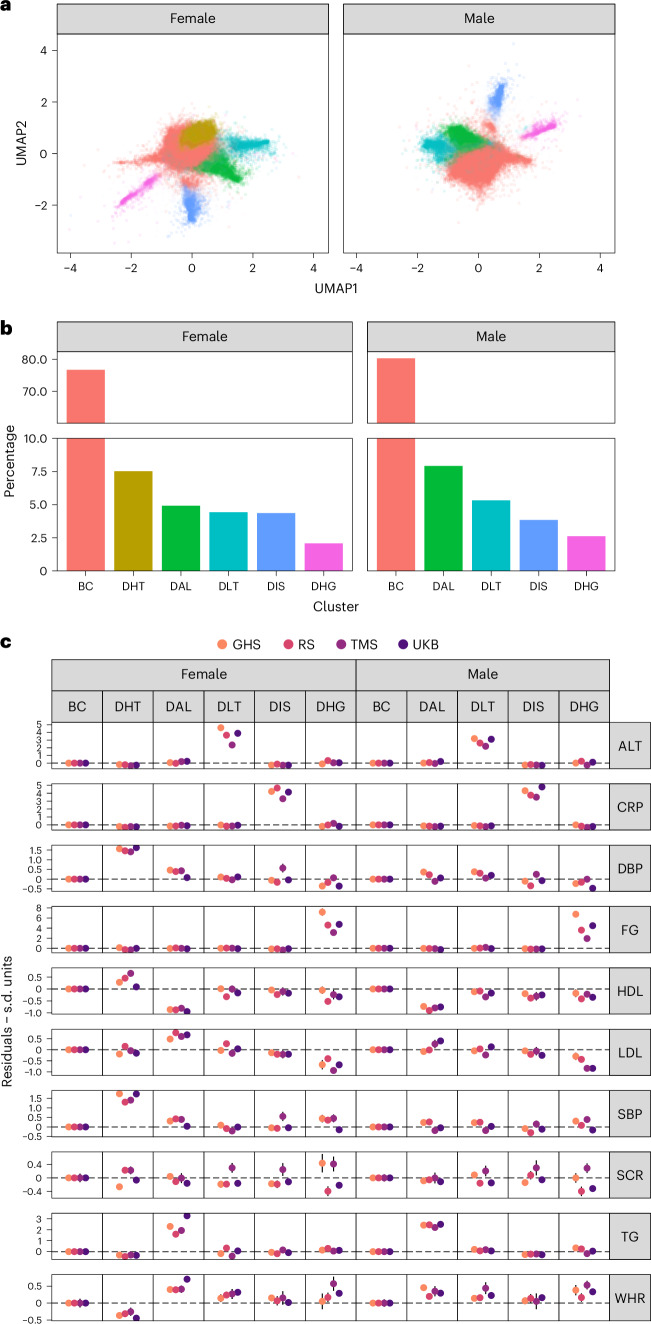
Characteristics of concordant and discordant profiles. Discordant profiles discovered in the UKB and robustly replicated across three independent cohorts. **a**, UMAP 2D projection. Colors denote profile allocations. **b**, Cluster weights. **c**, Forest plot of average biomarker residuals characterizing each profile. Points and error bars represent estimates and 95% confidence intervals of average residual values of each biomarker. The dashed line represents a residual of 0. Female sample sizes: UKB = 77,207; TMS = 1,542; RS = 5,704; GHS = 7,301. Male sample sizes: UKB = 67,904; TMS = 1,633; RS = 4,289; GHS = 7,353. DBP, diastolic blood pressure; SBP, systolic blood pressure; SCR, serum creatinine; WHR, waist-to-hip ratio.

**Extended Data Fig. 1:**
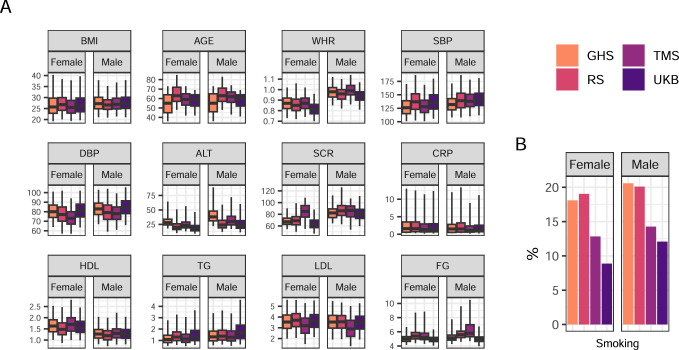
Distribution of BMI and biomarkers per cohort. **a**. Boxplots showing the distribution of continuous variables across cohorts. In each boxplot, the centre represents the median, the bounds of the box represent the interquartile range, and the whiskers represent the 2.5 and 97.5 percentiles. **b**. Proportion of current smokers in each cohort. Female sample sizes: UKB = 77,207; TMS = 1,542; RS = 5,704; GHS = 7,301. Male sample sizes: UKB = 67,904; TMS = 1,633; RS = 4,289; GHS = 7,353. WHR: waist-to-hip ratio. SBP: systolic blood pressure. DBP: diastolic blood pressure. ALT: alanine transaminase. SCR: serum creatinine. CRP: C-reactive protein. HDL: high-density lipoprotein. TG: triglycerides. LDL: low-density lipoprotein. FG: fasting glucose.

**Extended Data Fig. 2:**
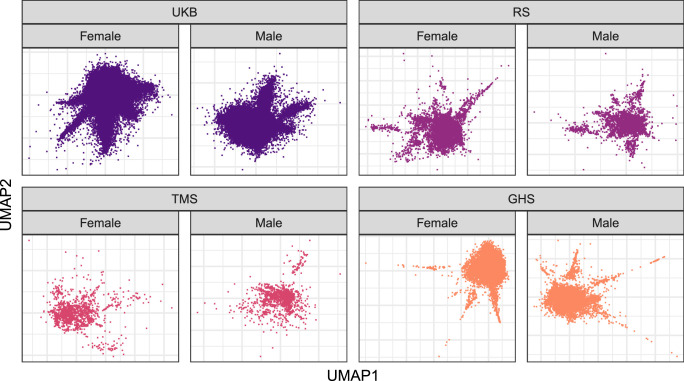
UMAP projections of BMI-biomarker discordance across cohorts. Two-dimension projections derived from the UMAP algorithm. GHS: Gutenberg Health Study, TMS: Maastricht Study, RS: Rotterdam Study, UKB: UK Biobank.

**Extended Data Fig. 3:**
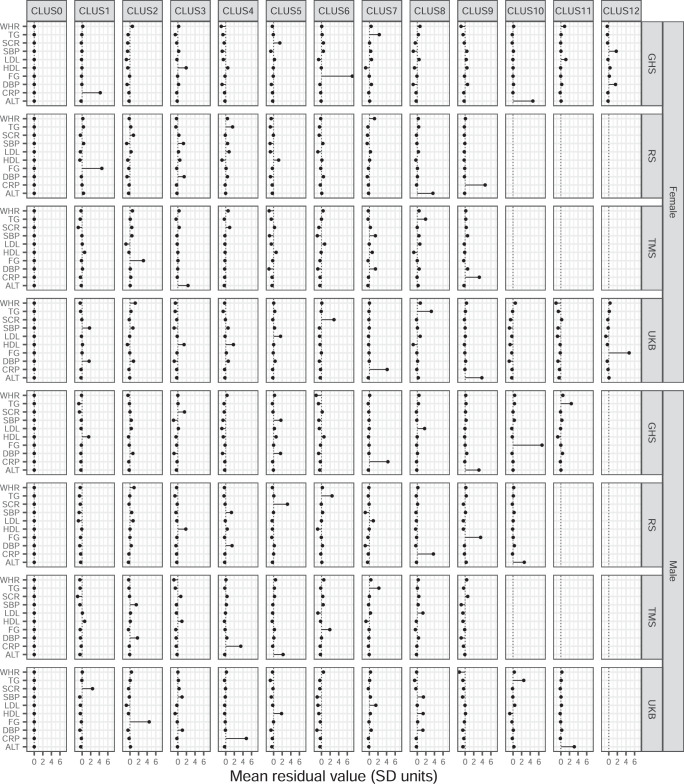
Discordant profile centres across cohorts. These centres were obtained by running the clustering approach on the 4 cohorts separately. Points represent average residual values within each profile, and the lines depict the distance from the null residual value. WHR: waist-to-hip ratio. TG: triglycerides. SCR: serum creatinine. SBP: systolic blood pressure. LDL: low-density lipoprotein. HDL: high-density lipoprotein. FG: fasting glucose. DBP: diastolic blood pressure. CRP: C-reactive protein. ALT: alanine transaminase.

**Extended Data Fig. 4:**
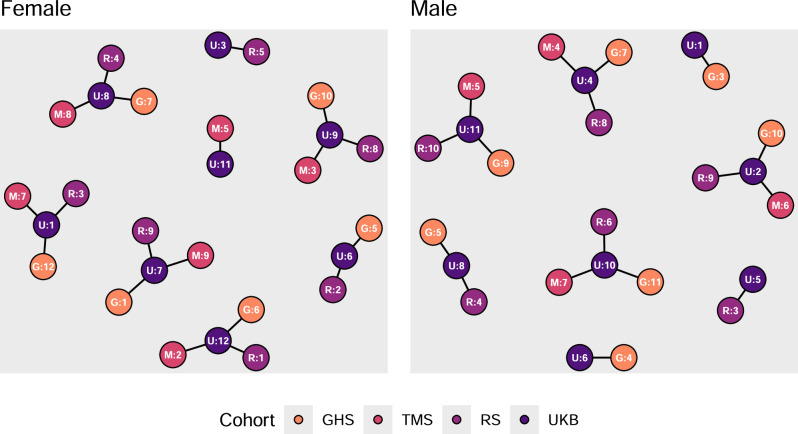
Validation of discordant profiles identified in UK Biobank. Nodes are clusters found by running our clustering algorithm in each cohort separately. The numbering is the same as in Extended Data Figure 3. Edges are drawn between a cluster from the UKB model and a cluster from another cohort if the subset of individuals with high probability to be allocated to the UKB cluster (>80%) have also a high median probability (>80%) to be allocated to the cluster from the other cohort. Only UKB clusters with edges to 3 clusters from each of the other cohort were considered replicated. GHS: Gutenberg Health Study, TMS: Maastricht Study, RS: Rotterdam Study, UKB: UK Biobank.

To further validate the identified subgroups, we conducted the same analysis in three independent large population-based cohorts: the Maastricht Study (TMS, *N* = 3,175), the Rotterdam Study (RS, *N* = 9,993) and the Gutenberg Health Study (GHS, *N* = 14,654). Baseline characteristics are shown in Supplementary Tables 1–3 and Extended Data Fig. 1. The effects of BMI–biomarker relationships within each cohort are shown in Supplementary Fig. 5 and Supplementary Table 4. In all UMAP projections obtained, we observed a pattern of ‘spikes’ deviating from a central ‘cloud’ where most individuals were located, which corresponded to individuals with BMI–biomarker discordance (Extended Data Fig. 2). After determining which profiles were consistently replicated across all cohorts (Methods and Extended Data Figs. 3 and 4), we obtained a final partition that included a concordant profile and four discordant profiles in men and five in women (Fig. 2a,b). The median allocation probability for an individual’s highest scoring profile exceeded 90% (interquartile range = 89–99%), and the relative entropy of this final partition was 0.85 for men and 0.88 for women, suggesting that the profiles in these models were well discriminated.

To assess the quality of the partition, we compared the final partition with partitions derived from diverse classes of flexible probabilistic clustering algorithms applied to the deviation data. We performed centroid-based (Gaussian mixture), boundary-based (archetypes) and density-based (HDBSCAN) algorithms. The best partitions obtained from these other algorithms had lower relative entropies in comparison with our final partition (Supplementary Table 5) and were unable to accurately identify the subgroups we observed in the UMAP projections (Supplementary Figs. 6–14).

We observed that discordant profiles conveyed multivariate profiles of discordance. They differed from the concordant profile in the values of multiple biomarkers (Supplementary Figs. 15–17 and Supplementary Tables 6 and 7), the magnitude to which the biomarkers deviated from the expected value given their BMI (Fig. 2c) and the correlation among these deviations (Supplementary Fig. 18). Most individuals (~80%) had a predominantly concordant phenotypic profile, with biomarkers within the normal distribution of the expected values for their BMIs, which we termed the ‘baseline concordant’ (BC) profile (Fig. 2b and Supplementary Table 8). Approximately 8% of women displayed a discordant hypertensive profile (DHT), with blood pressure values greater than expected for their BMIs. This profile was not replicated in men. Around 5% of women and 7% of men showed a discordant adverse lipid profile (DAL), characterized by higher TG, lower HDL and higher LDL than expected for their BMIs. Profiles of discordant liver transaminase (DLT) and discordant inflammatory state (DIS), respectively characterized by higher-than-expected ALT and CRP, were each observed in 4–5% of individuals in both sexes. Lastly, about 2.5% of individuals had a discordant hyperglycemic profile (DHG), with discordantly high FG levels, correlating with discordantly lower LDL levels. Notably, individuals with a concordant profile formed a less tightly connected subgroup compared with the discordant profiles, as measured by the transitivity index (Supplementary Table 9), suggesting that discordant profiles exhibit more cohesive biomarker patterns. To better understand how biomarker variation corresponds to discordant profile probabilities, we show in Supplementary Table 10 the biomarker values corresponding to varying levels of discordant profile probabilities for an individual with a fixed age (55 years) and BMI (30 kg/m^−2^) who do not smoke.

**Fig. 3:**
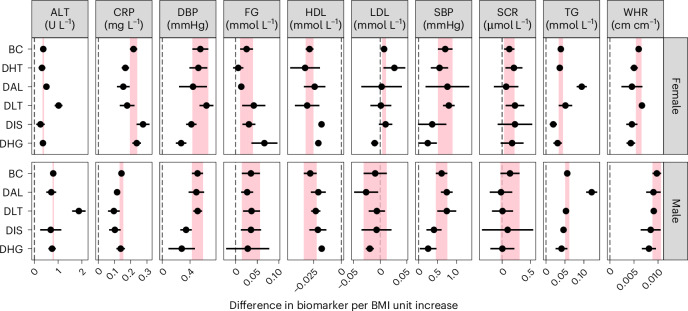
Estimated biomarker change per BMI unit increase within each profile. Pooled estimates and 95% CI of the change in each biomarker corresponding to a BMI unit increase within each profile. Estimates were derived using random effects meta-analysis across studies. Areas shaded in pink correspond to the CI around the estimate of the BC profile. The dashed line represents the null association. Female sample size = 91,754; male sample size = 81,178.

We found that discordant and concordant profiles also differed in their BMI–biomarker relationships (that is, how biomarkers change when BMI increases, using profile allocation probabilities as regression weights to obtain profile-specific estimates). For example, we found that in both the male and female DAL profiles, a unit increase in BMI had around twice the effect on TG compared with the effect observed in BC (Fig. 3, Supplementary Fig. 19 and Supplementary Table 11).

We found that an overall favorable biomarker discordance (that is, all biomarkers at lower levels than expected for a given BMI, except HDL at higher levels) was rare (0.92% females, 0.81% males) and this was not distinct from the concordant profile (Supplementary Fig. 20), implying that this is part of the normal distribution of concordance.

### Discordant profiles and cardiometabolic disease prevalence

**Fig. 4:**
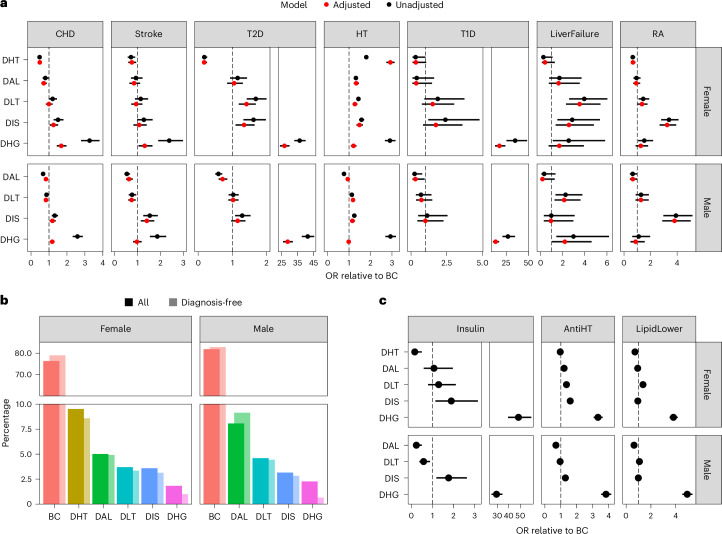
Association of discordant profiles with prevalent comorbidities and medication. **a**, OR and 95% CI of selected conditions in discordant profiles relative to the concordant profile, unadjusted and adjusted for medication (lipid-lowering, antidiabetic and antihypertensive). The dashed line represents the null association. **b**, OR and 95% CI of selected medications in discordant profiles relative to the concordant profile. **c**, Comparison of the proportions of concordant and discordant profiles in individuals without the selected conditions against all individuals in UKB. The dashed line represents the null association. Female sample size = 91,754; male sample size = 81,178. AntiHT, antihypertensives; HT, hypertension; LipidLower, lipid-lowering medication; RA, rheumatoid arthritis.

We estimated profile-specific prevalence of various cardiometabolic comorbidities associated with the biomarkers selected using allocation probabilities as weights (Fig. 4a and Supplementary Table 12). Whereas disease cases were predominantly of a concordant profile, disease prevalences in discordant and concordant profiles differed substantially from one another. For example, after a 5% false discovery rate (FDR) correction, women with a DHG profile were 3.26 times more likely to have suffered from coronary heart disease (CHD) compared with BC (95% confidence intervals (CI) 2.79, 3.82). There was also an enrichment of CHD cases in DIS compared with BC (odds ratio (OR) 1.50, 95% CI 1.25, 1.80). The same pattern of CHD enrichment was found in men (OR in DHG 2.59, 95% CI 2.32, 2.88; OR in DIS 1.32, 95% CI 1.16, 1.50). Notably, there were fewer cases of CHD in DAL compared with BC in both sexes (OR in women 0.79, 95% CI 0.64, 0.98; OR in men 0.67, 95% CI 0.60, 0.75). We also observed a depletion of CHD cases within the DHT profile in women (OR 0.47, 95% CI 0.39, 0.58).

Aside from the expected enrichment of cases of T2D in DHG compared with BC (>30 times greater prevalence than in the concordant profile in both sexes), the DIS and DLT profiles were also enriched in women with T2D (OR in DIS: 1.62, 95% CI 1.32, 1.98; OR in DLT: 1.68, 95% CI 1.42, 2.00). Conversely, the prevalence of T2D was lower in DAL than BC in men (OR 0.59, 95% CI 0.50, 0.70) but not in women (OR 1.15, 95% CI 0.93, 1.43). The prevalence of T2D was also lower in women classified as DHT than those classified as BC (OR 0.18, 95% CI 0.12, 0.26). The subset of individuals who were free of cardiometabolic conditions (CHD, stroke, type 1 diabetes (T1D), T2D, hypertension, liver failure, rheumatoid arthritis), had similar profile allocations, with the exception of lower DHG profile probabilities (Fig. 4b). We also assessed enrichment of the metabolic syndrome across profiles, using the World Health Organization criteria^15^ (Supplementary Fig. 21 and Supplementary Table 13). While most individuals with the metabolic syndrome had a concordant profile (>60%), we found statistically significant enrichment for individuals with the metabolic syndrome in discordant compared to the concordant profiles, particularly in DHG (more than 30-fold increase). Two exceptions were the DHT profile in women and the DAL profile in men, which were associated with lower prevalence of metabolic syndrome compared with the concordant profile.

We investigated medication use within each profile (Fig. 4c and Supplementary Table 14) and showed enrichment for insulin, antihypertensive and lipid-lowering therapy use in the DHG profile. For example, insulin therapy was >30 times more frequent in the discordant compared with the concordant profile. Fewer men with a DAL profile were medicated with lipid-lowering medication compared with the concordant profile (OR 0.64, 95% CI 0.59, 0.68). The observed disease associations were largely unmodified after adjustment for these medications, except for the OR estimates for the DHG profile, which were significantly attenuated (Fig. 4a).

### Discordant profiles and cardiometabolic disease incidence

To evaluate the effect of discordant profiles on future risk of disease, we used longitudinal data derived from the clinical records of up to 155,000 individuals from UKB, RS and GHS, who were free from the index disease at baseline (Supplementary Table 15). We first derived crude incidence estimates of MACE and T2D at 5–10 years of follow-up for each profile applying the same weighted approach as previously described (Supplementary Table 16). After multiple test correction, the DHG and DIS profiles were associated with higher risk of MACE compared with BC across sexes (10-year DHG relative risk (RR) 1.96, 95% CI 1.66, 2.31; 10-year DIS RR 1.46, 95% CI 1.25, 1.72). The female, but not the male, DAL profile was associated with higher MACE relative to BC (10-year female RR 1.40, 95% CI 1.21, 1.61; 10-year male RR 0.96, 95% CI 0.87, 1.07). In general, all discordant profiles were associated with higher risk of incident T2D compared with BC, particularly the DHG profile, which displayed a 6–13-fold increase in T2D risk. The only exception was the female DHT profile, which was associated with lower risk of T2D compared with BC (10-year RR 0.46, 95% CI 0.35, 0.60).

To assess the added clinical value of these profiles for MACE prediction, we added profile allocation probabilities to sex-specific survival models. The predictor variables in these models consisted of all the biomarkers used to produce the initial clustering partition, as well as all variables and interactions used in the current CVD risk stratification tool endorsed by the European Society of Cardiology (SCORE2)^16,17^. We also included in these models the baseline comorbidities we evaluated in Fig. 3a (see Supplementary Table 17 for variable list). Through the comparison of nested models, we showed that adding profile information improved the predictive ability of these prediction models in UKB, especially in men, as shown by significant likelihood ratio tests and difference in C-statistics (Table 1). The additional explained variation in MACE attributed to the profiles in UKB ranged from 1.4% to 5.4%. Although profile information explained additional variance in RS and GHS, the likelihood ratio tests were not statistically significant.

**Table 1:** Model comparison without and with profile allocation probabilities

Outcome	Follow-up time (years)	Sex	Cohort	LRT *P* value	5% FDR	Added variance explained (%)	C-statistic baseline model	C-statistic baseline + profiles	C-statistic difference	Difference *P* value	5% FDR
MACE	5	Female	UKB	3.63 × 10^−4^	True	2.31	0.733	0.735	0.002	0.075	False
MACE	5	Female	RS	0.997	False	0.16	0.768	0.769	0.001	0.399	False
MACE	5	Female	GHS	0.133	False	5.00	0.816	0.820	0.004	0.376	False
MACE	5	Male	UKB	2.70 × 10^−16^	True	5.44	0.704	0.709	0.005	3.33 × 10^−5^	True
MACE	5	Male	RS	0.530	False	1.78	0.721	0.721	0.000	0.953	False
MACE	5	Male	GHS	0.998	False	0.06	0.771	0.771	0.000	0.581	False
MACE	10	Female	UKB	5.23 × 10^−5^	True	1.36	0.726	0.728	0.002	0.035	True
MACE	10	Female	RS	0.886	False	0.43	0.763	0.763	0.000	9.62 × 10^−1^	False
MACE	10	Male	UKB	4.22 × 10^−20^	True	4.23	0.685	0.690	0.005	1.19 × 10^−6^	True
MACE	10	Male	RS	0.271	False	1.88	0.711	0.712	0.001	0.840	False
DM	5	Female	UKB	0.893	False	0.11	0.872	0.872	0.000	0.558	False
DM	5	Female	RS	3.80 × 10^−6^	True	10.79	0.811	0.822	0.011	0.043	False
DM	5	Female	GHS	1.41 × 10^−3^	True	8.97	0.806	0.814	0.008	0.126	False
DM	5	Male	UKB	0.872	False	0.07	0.841	0.841	0.000	0.439	False
DM	5	Male	RS	4.03 × 10^−4^	True	8.38	0.816	0.821	0.005	0.318	False
DM	5	Male	GHS	0.606	False	1.24	0.802	0.804	0.002	0.388	False
DM	10	Female	UKB	0.046	False	0.49	0.854	0.855	0.001	0.162	False
DM	10	Female	RS	3.92 × 10^−11^	True	12.83	0.796	0.808	0.012	0.005	True
DM	10	Male	UKB	0.048	False	0.38	0.821	0.822	0.001	0.001	True
DM	10	Male	RS	3.47 × 10^−8^	True	11.05	0.804	0.808	0.004	0.244	False

LRTs, fraction of additional variance explained and C-statistics comparing nested models with versus without discordant profile allocation probabilities. LRT *P* values are derived from the chi-square distribution with degrees of freedom equal to the number of additional parameters in the full compared with the reduced model. The variance of the difference in C-statistic considers the covariance of the C-statistics from the two models. The resulting *P* value is derived from a two-sided normal test. DM, diabetes mellitus; LRT, likelihood ratio test.

**Fig. 5:**
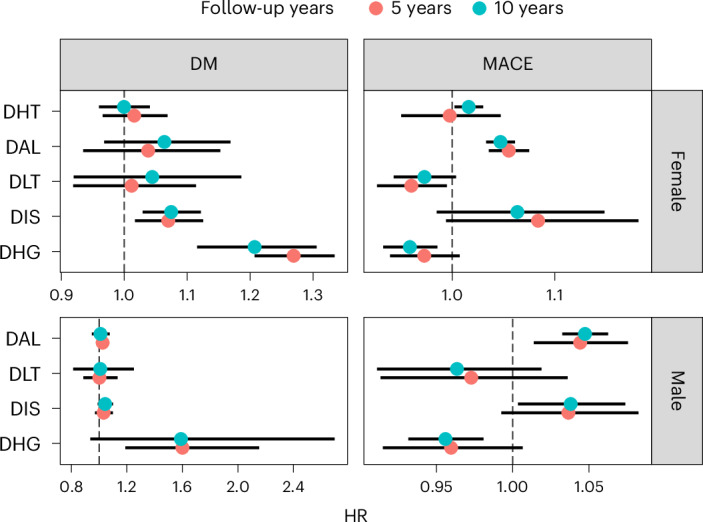
Hazard ratios of discordant profiles. HR estimates and 95% CI associated with shifting 10% probability from the concordant to each of the discordant clusters, derived from a random effects meta-analysis across cohorts. Pooled female sample sizes: MACE = 85,392; DM = 46,076. Pooled male sample sizes: MACE = 70,328; DM = 38,815.

Because an individual’s allocation probability for any given profile is determined by the biomarkers and BMI, discordant profile estimates from these survival models reflect complex interactions between profiles, biomarkers and BMI. These interactions modify the associations between biomarkers and risk, conditional on their specific profile of discordance with BMI (Supplementary Fig. 22 and Supplementary Table 18). These interaction estimates were also robust to regularization using the Lasso penalty (Supplementary Fig. 23). To better understand these profile estimates, we derived the expected change in risk of MACE in a disease-free, 55-year-old individual, with a BMI of 30 kg/m^−2^, when their profile allocation probability is raised by 10% for any given profile, with a corresponding decrease in the probability of having a concordant profile (Fig. 5, Supplementary Fig. 24 and Supplementary Table 19). After multiple test correction, an increased probability for the DAL profile was associated with higher risk of MACE compared with BC across sexes (10-year hazard ratio (HR) in women 1.04, 95% CI 1.03, 1.06; 10-year HR in men 1.05, 95% CI 1.04, 1.06). By contrast, increased probability for the DHG profile was associated with lower risk of MACE compared with BC (10-year HR in both sexes 0.95, 95% CI 0.93, 0.98).

Using the same approach, we determined the clinical value of the discordant profiles by estimating the 5- and 10-year risk of incident diabetes in individuals without diabetes. We found that adding profile information only marginally increased the variance explained in diabetes progression in UKB. However, in RS, where the median glucose values were higher, the fraction increased to 8–12% (likelihood ratio *P* < 0.001). After multiple test correction, only the female DHG profile remained associated with diabetes progression. A 10% increase in the probability of having a DHG profile at the expense of lowering the probability of having a concordant profile was associated with a 20–60% increase in risk of progressing to diabetes compared with individuals in BC.

**Fig. 6:**
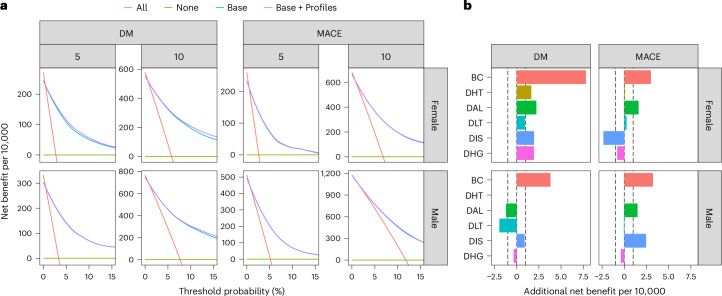
Decision curve analysis of discordant profiles. **a**, Decision curves comparing the net benefit of using various strategies at different thresholds of disease probability up to 15%. **b**, Distribution of gains in net benefit at threshold for intervention of 10% risk of disease at 10 years. Dashed vertical lines are unit gain and unit loss in net benefit per 10,000 individuals assessed. Base, initial prediction model incorporating baseline clinical data; Base + Profiles, second prediction model incorporating baseline clinical data and profile information.

We then evaluated the added net benefit of discordant profiles using decision curves to determine whether conducting interventions to prevent MACE is likely to be worthwhile (Fig. 6a and Supplementary Table 20)^18–20^. For this, we compared the prediction models created using only baseline data (including baseline biomarker values and other relevant clinical characteristics) with models that additionally incorporated profile estimations. Both models with and without discordant profile information generally outperformed default strategies of no intervention or universal intervention at various thresholds of disease probability up to 15%. At a threshold of a 10% 10-year MACE risk (traditionally used to determine statin initiation, and equivalent to accept intervening nine individuals without the disease (false positives) to prevent one event (true positive)), adding profile information yielded an average net benefit of 4 additional true positives and 37 additional true negatives per 10,000 men compared with the baseline model. To benchmark these values against a contemporary standard, we computed the additional net benefit of LDL, an established intervention target for MACE, over and above the predictive value of chronological age; the inclusion of LDL resulted in 5 additional true positives and 42 additional true negatives per 10,000 male individuals tested. Thus, discordancy and LDL can be considered of comparable value for the prediction of MACE. In women, adding discordant profile information did not yield any material net benefit.

Discordant profile information had the highest utility in determining women at risk of diabetes progression. Using a 10-year risk threshold of 10%, we found that discordant profile information led to a net benefit of 15 additional true positives and 135 additional true negatives per 10,000 women compared with the baseline model. In men, the additional net benefit was 4 additional true positives and 33 additional true negatives per 10,000 men.

We next examined how the benefits of adding discordant profile information were distributed across profiles (Fig. 6b). Net benefits in MACE were concentrated in the BC and DAL profiles in both men and women. Notably, we observed improvements in net benefit in the DIS profile for men, but in women net benefit declined. For diabetes progression, we observed improvements in net benefit across all profiles in women. Conversely, among men, we observed improvements only in the BC and DIS profiles.

### Discordance by ethnicity

We evaluated how the discordant profiles identified in European populations were distributed in British African and South Asian populations in UKB (*N*_African_ = 4,019, *N*_SouthAsians_ = 3,388; Supplementary Figs. 25 and 26 and Supplementary Table 21). South Asian individuals had around four times higher odds of having a DHG profile compared with people of European ancestry (female OR 3.87, 95% CI 3.13, 4.72; male OR 4.61, 95% CI 3.90, 5.41). We observed a similar enrichment of DHG in the African population, albeit of lesser magnitude (female OR 2.08, 95% CI 1.65, 2.59; male OR 2.54, 95% CI 2.05, 3.11). South Asian people were also more likely to have a DAL profile than people of European ancestry (female OR 1.79, 95% CI 1.49, 2.13, male OR 1.38, 95% CI 1.18, 1.60). Women of African and South Asian ancestry had higher probabilities than women of European ancestry of having a DIS profile (OR African 1.39, 95% CI 1.13, 1.68; OR South Asian 1.80, 95% CI 1.45, 2.20). Women of African ancestry also had higher DHT probabilities than European women (OR 1.25, 95% CI 1.09, 1.42).

We observed enrichment of diseases and medication use at baseline predominantly in the DHG profile compared with the BC profile, as seen in the European population (Supplementary Tables 22 and 23). In adjusted survival analyses including discordant profile information (Supplementary Table 24), we found that in South Asian men, a 10% higher probability of having a DAL profile was associated with higher 10-year MACE risk compared with a BC profile (HR 1.10, 95% CI 1.05, 1.15), whereas a 10% higher DHG probability was associated with lower risk (HR 0.84, 95% CI 0.74, 0.95), comparable with the findings in European men.

By contrast to European women, South Asian women with a 10% higher DHT probability had a higher 10-year MACE risk compared with the BC profile (HR 1.09, 95% CI 1.03, 1.16). A 10% higher DHG probability conveyed an especially high risk of diabetes progression at 10 years in African men (HR 1.68, 95% CI 1.21, 2.34), consistent with estimates in European men. By contrast, the risk of diabetes progression was not increased in women of African ancestry or in men or women of South Asian ancestry at a 10% DHG probability. The likelihood ratio tests comparing models with and without discordant profile information were not statistically significant after multiple test correction in any of these ethnic groups. Discordance independently enhanced MACE and T2D prediction in men of South Asian and African ancestry, respectively, at a nominal level of statistical significance (likelihood ratio test *P* < 0.05) (Supplementary Table 25).

## Discussion

The relationship between elevated BMI and other morbidities is highly heterogeneous, underscoring the inability of this simple measure to adequately characterize the pathophysiological complexities of obesity. Here, we deconvoluted this heterogeneity using unsupervised clustering to identify five phenotypic profiles that are defined by atypical relationships between BMI and risk biomarkers. In turn, these clusters convey risk profiles for CVD and diabetes that differ markedly from those seen in the more common concordant profile. These results robustly replicated across four independent population-based cohorts. Collectively, these discordant clusters characterize ~20% of the general population and improve the precision and accuracy of CVD and T2D risk prediction to a similar degree as contemporary clinical risk markers such as LDL. Using this discordancy approach would lead to a net benefit of 37–135 unnecessary interventions avoided, and appropriate interventions initiated in an additional 4–15 patients for every 10,000 individuals tested.

We show, for instance, that MACE prediction improves when discordance between lipid fractions and BMI (that is, DAL) is incorporated into standard prediction models. DAL resembles the phenotypic characteristics of familial combined hyperlipidemia, where a dearth of adipose tissue drives cardiogenic dyslipidemia^21–23^. Diagnosis of familial combined hyperlipidemia is challenging because of its heterogeneous presentation, often requiring extensive testing of the proband and their family members^23^. We found that individuals with a DAL profile had a lower prevalence of MACE at baseline and were less often prescribed medication, indicating that determining DAL in these individuals might aid early risk stratification and prevention. Importantly, the DAL profile describes a subgroup of relatively lean people at elevated cardiovascular risk.

Two discordant profiles (DHG, DLT) predisposed lower MACE incidence, despite being characterized by elevated biomarkers that are typically considered cardiogenic. The DHG profile more frequently included people with multimorbidity, including a higher prevalence and incidence of diabetes. In models adjusted for these comorbidities, individuals with the DHG profile who were disease-free at baseline were less likely to develop MACE than their counterparts with concordant profiles. This may be because glycemia and LDL concentrations are inversely related in this profile. This inverse relationship, in which T2D risk is elevated, is also observed in people with a genetic predisposition to low LDL concentrations^24^.

Similarly, the DLT profile, characterized by higher discordant ALT, had no association with diabetes progression and conveyed lower MACE risk compared with the concordant profile. Higher blood concentrations of ALT and other markers of liver dysfunction have been linked to elevated CVD risk, although this risk profile is usually concomitant with obesity^25,26^, as is the case with the concordant profile. However, the relationship between ALT and CVD risk is nonlinear, with inverse associations between ALT and CVD risk observed when ALT levels are within the normal range^27,28^, as well as in alcoholic and nonalcoholic liver disease^29^. The relationship between ALT concentrations and CVD risk also appears to be modified by diabetes, with ALT positively associated with CVD mortality in the presence of diabetes and inversely associated with CVD mortality when diabetes is absent^30^, consistent with the DLT risk profile described here.

No DHT profile was observed in males. Although hypertension and obesity are more prevalent in males than in females, BMI is reportedly a stronger risk factor for hypertension in females than in males^31^. This may be in part attributable to the effects of menopause and hormone-replacement therapy^32^.

Our analyses show that accuracy can be improved when discordancy variables are included in prediction models. Nevertheless, although the discordant profiles conveyed similar estimates of MACE and diabetes risk in men and women, predictive accuracy sometimes varies by sex. For example, in discordant women, adding the DIS profile to MACE prediction models diminished accuracy, whereas in discordant men, accuracy improved. This may reflect sex-specific differences in cardiometabolic risk profiles; in women, for example, CRP concentrations are generally higher and the relationships of CRP with adipose mass and adipose distribution are typically stronger than in men^33^. Conversely, including the discordant profiles in models predicting diabetes progression improved predictive accuracy in women more than in men. These differences are consistent with published analyses, in which diabetes prediction accuracy is generally higher in women than in men, especially when models include anthropometric variables^34^. It is important to acknowledge that we did not formally test whether the profile effects differed by sex, for example by combining data for both sexes and testing for sex by profile interactions. This is because profiles were estimated separately for each sex. Consequently, the scale of discordance is different by sex.

Our approach to defining discordant subgroups applies nonlinear clustering techniques to large datasets, describing the distribution of multivariate data without the constraints of linear assumptions. Similar techniques have been used elsewhere to help resolve clinical heterogeneity in new-onset T2D^35^. Although classification methods have often been used to resolve disease heterogeneity, doing so frequently disregards intraprofile heterogeneity, interprofile overlapping and misclassification^36^. The partitioning algorithm used here addresses these limitations by allocating profile probabilities based on the specific phenotypic combinations. This approach enables the effects of discordances to be more precisely estimated, even within the concordant profile. This allows for the continuous nature of risk to be captured, incorporating both the BMI-independent effects of biomarkers and the effects of BMI-discordance^37^.

Several limitations should be acknowledged. First, our findings are based on a limited set of biomarkers and the cohorts are homogeneous (35–75-year-old adults of European ancestry), also it is not known whether these findings will transfer adequately to other populations. Second, although our study included four large independent cohorts and the profiles identified were successfully replicated across all cohorts, the proportion of participants with the discordant profiles was small, which likely limited statistical power for the discovery and replication analyses performed here. Better separation between subgroups might be possible if a more comprehensive biomarker set was to be included in the clustering analysis. Third, the data used to derive the clusters were cross-sectional. Thus, some biomarker levels will have been imprecisely estimated owing to regression to the mean. This may have further impeded cluster discovery and replication^38^. Fourth, our study relied on clinical records to ascertain MACE and diabetes incidence, which may lead to an underestimation or overestimation of risk. Fifth, although we included in our analysis conditions that are commonly associated with altered levels of the biomarkers selected, many other conditions and medications (for example, mental health conditions, thyroid conditions, steroids) can alter BMI–biomarker associations. Achieving full adjustment for all these variables is therefore challenging. However, because initial assessments typically rely on the selected biomarkers, examining their discordance with BMI can serve as a valuable initial approach for risk stratification.

In conclusion, we identified five distinct phenotypic profiles exhibiting diverse relationships between BMI and cardiometabolic biomarkers and varying degrees of CVD and diabetes risk. These analyses help resolve some of the substantial heterogeneity in the relationship between BMI and disease risk. Incorporating phenotypic discordance into contemporary risk scores enhances the prediction of MACE and diabetes progression in the general population.

## Methods

### Study cohorts

#### The UK Biobank

The UKB is a large prospective cohort that recruited more than 500,000 adults (aged 37–73 years) during 2006–2010 (ref. ^39^). Participants provided comprehensive demographic, health, biological, cognitive, social, lifestyle, mental and well-being data. This specific analysis was approved by the UKB research committee (approval ID: 57232). Longitudinal outcome data for up to 10 years follow-up were extracted from clinical and mortality records.

#### The Maastricht Study

The MS is an observational prospective population-based cohort study. The rationale and methodology have been described previously^40^. In brief, the study focuses on the etiology, pathophysiology, complications and comorbidities of type 2 diabetes mellitus (T2DM) and deployed an extensive phenotyping protocol. Eligible for participation were all individuals aged between 40 and 75 years living in the southern part of the Netherlands. Participants were recruited through mass media campaigns and from municipal registries and the regional Diabetes Patient Registry via mailings. Recruitment was stratified according to known T2DM status, with an oversampling of individuals with T2DM, for reasons of efficiency. The current report includes cross-sectional data from the first 7,689 participants, who completed the baseline survey between November 2010 and December 2017. The examinations of each participant were performed within a time window of three months. The study has been approved by the institutional medical ethical committee (NL31329.068.10) and the Minister of Health, Welfare and Sports of The Netherlands (Permit 131088-105234-PG). All participants gave written informed consent.

#### The Rotterdam Study

The RS is a population-based cohort study conducted in the Ommoord district of Rotterdam, The Netherlands, with the primary objective of assessing common diseases among the elderly population. The study, which has been extensively documented,^41^ recruited 7,983 individuals aged 55 years or older for the initial RS-I cohort in 1990. Subsequently, in 2000, the RS-II cohort was expanded by 3,011 participants who either relocated to the study area or reached the age of 55. The cohort was further extended with 3,932 participants aged 45 years or older (RS-III). Baseline evaluations were conducted through home interviews and comprehensive physical examinations at the time of recruitment, followed by subsequent visits every 3–4 years for follow-up assessments. We included longitudinal outcome data up to 10 years after recruitment.

#### The Gutenberg Health Study

The GHS is a prospective and observational adult population-based cohort study in the Mainz–Bingen region of Rhine–Palatine in Germany. The study sample consisted of 15,010 participants aged 35–74 years who were enrolled at their baseline examination between 2007 and 2012. Each study participant underwent a comprehensive standardized clinical and laboratory examination at enrollment. We included follow-up outcome data up to 5 years after recruitment. More detailed information on the study design has been published before^42^.

### Statistical analysis

#### Data preparation

We included 13 biomarkers: FG concentrations in mmol l^−1^; lipid fraction (HDL, LDL, TG) concentrations in mmol l^−1^; systolic and diastolic blood pressures in mmHg; serum creatinine concentrations in μmol l^−1^; ALT concentrations in U l^−1^, CRP concentrations in mg l^−1^; waist-to-hip ratio in cm cm^−1^; age in years; current smoking status (1 for yes, 0 for no); and sex (male, female) from all cohorts. Variable units were converted to a common value where necessary. No BMI threshold was applied. From the UKB, TMS and GHS, we included only complete sets of all the biomarkers considered in the clustering analyses. RS included individuals for whom some biomarker values were missing (<10% missing), which were imputed using the Multiple Random Forest Regression Imputation method from the R package mice (v.3.16.0)^43^. Values >5 s.d. units from the mean were deemed to be erroneous and were consequently removed before the main analysis^44^. Owing to the established gender differences between BMI, some biomarkers and diabetes/CVD risk, all downstream analyses were stratified by sex.

#### Phenotypic discordance with BMI

We estimated the age and current smoking-adjusted associations in all selected biomarkers per unit increase in BMI by taking the residuals of a linear model with the respective biomarker as outcome and age and smoking status as the sole covariates. We then calculated the difference between expected and observed values, which were centered and scaled to have a mean of zero and unit standard deviation. To evaluate the proportion of individuals whose biomarker values deviate substantially from the expected given their BMI, we measured the squared Mahalanobis distance of every individual to a multivariate normal distribution around the expected values^45^. Because the squared Mahalanobis distance follows a chi-square distribution, we converted these distances to *P* values and assessed the proportion of individuals with *P* values above the critical threshold of 0.05 (expected proportion 5%). We compared the observed proportion with those expected using a binomial test (*P* < 0.05 was considered statistically significant).

#### UMAP projection and profile identification

We projected individual deviations in two dimensions using the umap function implemented in the R package uwot v.0.1.16 (ref. ^14^). We configured the number of nearest neighbors (nn) in each cohort as a function of sample size through the equation:

$${\mathrm{nn}}=\max (10,10+15{\times} ({\mathrm{log}}_{10}\left({N}_{{{\mathrm{total}}}}\right)-4))$$

in which *N*_total_ represents the total number of participants in each cohort. In addition, we set the ‘binary_edge_weights’ parameter of this function as true, ensuring that all nonzero edge weights in the graph are set to 1. Both configurations ultimately implement PacMAP, a modification of UMAP that better preserves the global and local structure from the high-dimensional space in the projection^46^. We also set the ‘dens_scale’ parameter to 1, which additionally implements densMAP, another modification of UMAP that improves preservation of the density (closely connected individuals will appear closer in denser areas in the projection)^47^. To find subgroups, we used the proximity network on which this projection is based. We first used the leading eigen vector algorithm^48^ to find stable initial seeds to subsequently run the Leiden algorithm, using the implementations available in the R package igraph v.2.0.2. The Leiden algorithm is designed to enhance community detection in large networks, by ensuring that identified communities are well connected. Through three phases, local moving of nodes, refinement of the partition and aggregation of the network, the algorithm guarantees connectivity, convergence to locally optimal assignments^49^, which we iterated more than 500 times to identify strongly interconnected regions while optimizing the modularity criterion. This resulted in hard partitions, where individuals are assigned to a single cluster. We then calculated for every individual the normalized eigen centrality scores for their respective clusters, which measures its importance within the cluster. We used these scores as weights to calculate the center and covariance matrix of each cluster, which were part of the Gaussian mixture distribution. Clusters in the center of the projection (where residual values are closer to 0) were less stable between iterations than those at the boundaries. To address this issue, we introduced a ‘concordant’ distribution of residuals in the Gaussian mixture calculation, represented by a zero mean and identity covariance matrix. As a result, individuals with a discordant profile that is insufficiently separated from the concordant profile would have similar probabilities for both profiles, and their allocation would therefore not be replicated (see ‘Profile replication’ section below). These individuals would instead be included in the concordant profile, enhancing the quality of the final partition^50^. We kept centers and covariance matrices fixed, and estimated the weights of each cluster, which represent their respective population proportions. The resulting partition included concordant and discordant profiles and every individual had a probability score for each profile, with the total probability scores equaling 1.

#### Profile replication

To assess the validity of the partitions identified in UKB, we ran the same pipeline of network construction, 2D visualization and clustering in TMS, RS and GHS, with the parameters as described above, and compared their results with UKB. We assessed whether individuals allocated to a profile in the original model from the UKB with high certainty (that is, a probability >80%) also had a similar median probability of being allocated to a profile found in any of the other three ‘validation’ cohorts (again, with a probability >80%). We considered a profile as having been replicated if this condition was met in all three validation cohorts, which ensured that only clusters represented in all three cohorts were included in the final model. We then readjusted the weights for each profile and focused all downstream analyses on these latter replicated clusters. The clusters were named according to the average residuals of all biomarkers.

#### Connectivity within profiles and quality of partitions

We assessed the connectivity of individuals within each profile by first labeling individuals according to the highest probability of allocation to any of the profiles. We then extracted the corresponding subgraphs for each profile from the UMAP graph and calculated the global transitivity index for each profile. This index measures the probability of two individuals who are connected to a common third individual also being directly connected to each other (a measure of how frequently ‘the friend of my friend is my friend’).

To assess cluster separation quality, we used UKB data to calculate the relative entropy of the final partition, also known as the Kullback–Leibler divergence, a measure derived from information theory^51^. This measure takes values from 0 to 1, indicating either identical probability distributions of all profiles (that is, equal probabilities to all profiles for every participant) or complete cluster separation (no overlap between clusters).

#### Comparison to other clustering algorithms

To compare the quality of the partition produced by our pipeline, we compared the final partition with the best results obtained by applying three clustering algorithms on the deviations from BMI-expectations in UKB, each with distinct underlying assumptions. We fitted a Gaussian mixture model directly to the deviation data (centroid-based), which looks for a mixture of multivariate distributions (each with its own center, covariance matrix and weight) that best describes the data^52^. We also fitted an archetypal model (boundary-based), which looks for the best combination of extreme points that enclose and summarize the data^53^. Both algorithms optimize the variance explained and are penalized by the number of clusters. In addition, we fitted an HDBSCAN model (density-based), which finds a set of stable dense ‘regions’ within the data, without constraining cluster shape, each with a minimum cluster size or density, which is controlled by the ‘minPts’ argument^54^. We fitted multiple models of the three algorithms by varying the number of clusters (Gaussian mixture and archetypal models, from 1 to 20) and minPts (various values from 5 to 1,000), and then selected the best solution based on the elbow method. We then assessed the relative entropy of the best solutions and compared these values to those obtained from our final partition.

#### Profile-specific estimates

We derived BMI–biomarker associations for each profile using linear regression where each individual was weighted by the respective profile probabilities. We calculated weighted means, standard deviations, medians and interquartile ranges for all biomarkers within each profile. Similarly, to derive profile-specific prevalences, we multiplied each case by the respective profile probability, and then divided by the sum of the probabilities for that profile. For incidence, the denominator was the sum of the product between the follow-up times and the profile probability for each individual. We calculated incidences of MACE and diabetes progression at 5 and 10 years after recruitment (definitions in Supplementary Table 26 and description of follow-up data provided in Supplementary Table 15). Using these prevalence and incidence estimates, we calculated ORs and RRs comparing each discordant profile to the concordant using binomial and Poisson regressions. We compute study-specific estimates as well as overall estimates using fixed and random effects models^55^. We reported the random effects models, as we found these were more conservative than the former.

#### Added value of profiles in prediction

To assess the added value of discordant profile information in prediction, we compare the performance of nested models with and without incorporating information of discordant profiles.

##### Profile allocation probability transformation

The use of profile allocation probabilities as predictors in regression renders models unidentifiable because of the sum-to-1 constraint. To address this issue, we applied the log-contrast framework, frequently used in compositional data analysis^37^. Under this framework, sum-to-1 predictors are incorporated in a regression model by constraining the sum of the corresponding effect estimates to be zero. We applied this by selecting the concordant profile as the reference profile and for all individuals we divided their discordant allocation probabilities by the concordant probability. The logarithm of these quotients can then be used as predictors, satisfying the aforementioned constraints.

##### Nested Cox regressions

The risk associated with profile allocations was evaluated by using nested Cox proportional hazard models for MACE and T2D. For each outcome, there were two nested models: a basic model that includes a variety of risk factors, and an alternative model that includes all the risk factors from the base model and additionally incorporates discordant profile allocation probabilities in the form of log ratios. The estimation of outcome incidence was conducted by considering two follow-up periods: 10 years and 5 years.

For MACE, all models encompassed predictors featured in SCORE2, a cardiovascular risk stratification score endorsed by the European Society of Cardiology^16^. We utilized a version of SCORE2 validated in diabetic populations^17^. We added biomarkers that were not considered in SCORE2 but were part of our clustering analysis, as well as conditions and medications that are commonly associated with the biomarkers selected. The full list of covariates in these models is shown in Supplementary Table 17. The MACE prediction models excluded all participants with prevalent CHD, peripheral arterial disease and stroke.

The nested Cox models for predicting the incidence of T2D included all the variables that we used in our clustering analysis. Because these models included FG, the model with discordant profiles captures the added value of discordance beyond the current level of glycemia. The full list of covariates in these models is also shown in Supplementary Table 17. To fit these models, we excluded participants with pre-existing T2D or T1D, as well as those who were on insulin and/or antidiabetes medications.

##### Regularized Cox regression

To help ensure that low event rates and high dimensionality are not skewing our key findings, we refitted the models in UKB using the Lasso penalty, which shrinks uninformative estimates towards zero. The optimal penalty value was selected based on the lowest deviance using 10-fold cross-validation, performed with the glmnet package in R^56^.

##### Comparison of nested Cox regressions

To compare the nested models within each cohort, we used likelihood ratio tests^57^ and difference in C-statistics^58^. We quantified the fraction of additional variance explained by discordant profiles as a percentage of the total variance explained using the model likelihoods, a method that is not affected by arbitrary threshold selections^57,59^. However, given that thresholds are needed to decide when to intervene in clinical practice, we investigated the utility of adding discordance information to traditional risk factors using decision curve analyses^18^. Model performance was evaluated at various disease probability thresholds for intervention, which represent the weight given to identifying a true positive in terms of false positives, as well as to identifying a true negative in terms of false negatives. Net benefit (in terms of true positives) and net interventions avoided (in terms of true negatives) provided by the models were calculated within each study based on these weights, and then averaged across studies^19^. Profile-specific decision curves were also derived by recomputing each calculation using each profile’s weights.

##### Risk estimates for discordant profiles

As with any regression, the effect estimate of each profile in the models represents the change in the outcome expected from increasing the log ratio of one discordant profile unit while keeping the other log ratios (and other covariates) constant. Because of the sum-to-1 constraint, this is equivalent to increasing the probability of a discordant profile by a certain factor (because we are using the natural logarithm, the factor is the square root of e) while decreasing all other profiles by the same factor, which effectively keeps the other log ratios constant. However, a change in the probability of a certain discordant profile inevitably carries changes not only in the other profile probabilities, but also in the biomarkers, and vice versa. In this context, discordant log ratios represent interaction terms that modify the relationship between biomarkers and disease events, conditional on their pattern of discordance with BMI. Hence, to correctly estimate the effect of a shift in the probability distribution from the concordant to a specific discordant profile, while keeping the other discordant profiles fixed at their population value, we included all the changes, both in biomarker and discordant log-ratio terms, that would correspond to this shift. Study-specific estimates were computed and then pooled using fixed and random effects meta-analyses^60^.

#### Discordance by ethnicity

We calculated profile probabilities in African (*N* = 4,019) and South Asian (*N* = 3,388) individuals ascertained at first assessment in UKB, and then compared the probabilities for each profile with those obtained in the European subset using binomial regression. We used the same approaches outlined above to calculate enrichment of diseases and medications, and risk of MACE and diabetes progression within each profile.

### Inclusion and ethics

All collaborators of this study who have fulfilled the criteria for authorship required by Nature Portfolio journals have been included as authors, as their participation was essential for the design and implementation of the study. Roles and responsibilities were agreed among collaborators ahead of the research. This work includes findings that are locally relevant, which have been determined in collaboration with local partners. This research was not severely restricted or prohibited in the setting of the researchers, and does not result in stigmatization, incrimination, discrimination or personal risk to participants. Local and regional research relevant to our study was considered in citations.

### Reporting summary

Further information on research design is available in the Nature Portfolio Reporting Summary linked to this article.

## Online content

Any methods, additional references, Nature Portfolio reporting summaries, source data, extended data, supplementary information, acknowledgements, peer review information; details of author contributions and competing interests; and statements of data and code availability are available at 10.1038/s41591-024-03299-7.

## Supplementary information

Supplementary Information Supplementary Figs. 1–26.

Reporting Summary

Supplementary Tables 1–26.

## Data Availability

UKB data are available through a procedure described at http://www.ukbiobank.ac.uk/using-the-resource/, where timeframe information can also be found. Restrictions apply to the availability of TMS data, which were used under license for the current study. Data are, however, available from the authors upon reasonable request and with permission of TMS management team. Timelines and conditions can be found at https://www.demaastrichtstudie.nl/research/data-guidelines. Access to RS can be requested through the management team (secretariat.epi@erasmusmc.nl), which has a protocol for approving data requests. Because of restrictions based on privacy regulations and informed consent of the participants, data cannot be made freely available in a public repository. More information can be found at https://www.erasmusmc.nl/en/research/core-facilities/ergo-the-rotterdam-study. Data from GHS are not publicly available because this is not covered by the informed consent of participants. However, access to the data in the local database is possible upon reasonable request according to the ethics vote. Interested scientists can make their requests to the Gutenberg Health Study Steering Committee (e-mail: ed.zniam-shg@ofni). More information can be found at http://www.gutenberghealthstudy.org/.

## Code availability

All analyses were performed in programming language R v.4.2 (https://www.r-project.org/). The scripts used can be found at https://github.com/danielcoral/SOPHIA_Cross_Sectional.
