# Author Correction: Subclassification of obesity for precision prediction of cardiometabolic diseases

**DOI:** 10.1038/s41591-024-03403-x

**Published:** 2024-11-21

**Authors:** Daniel E. Coral, Femke Smit, Ali Farzaneh, Alexander Gieswinkel, Juan Fernandez Tajes, Thomas Sparsø, Carl Delfin, Pierre Bauvin, Kan Wang, Marinella Temprosa, Diederik De Cock, Jordi Blanch, José Manuel Fernández-Real, Rafael Ramos, M. Kamran Ikram, Maria F. Gomez, Maryam Kavousi, Marina Panova-Noeva, Philipp S. Wild, Carla van der Kallen, Michiel Adriaens, Marleen van Greevenbroek, Ilja Arts, Carel Le Roux, Fariba Ahmadizar, Timothy M. Frayling, Giuseppe N. Giordano, Ewan R. Pearson, Paul W. Franks

**Affiliations:** 1 Genetic and Molecular Epidemiology Unit, Lund University Diabetes Centre, Department of Clinical Science, Lund University, Helsingborg, Sweden; 2 Maastricht Centre for Systems Biology (MaCSBio), Maastricht University, Maastricht, The Netherlands; 3 Department of Epidemiology, Erasmus MC University Medical Center, Rotterdam, The Netherlands; 4 Preventive Cardiology and Preventive Medicine, Center for Cardiology, University Medical Center of the Johannes Gutenberg-University Mainz, Mainz, Germany; 5 Department of Pharmacometrics, Novo Nordisk A/S, Søborg, Denmark; 6 Université de Lille, Inserm, CHU Lille, Institut Pasteur de Lille, U1190-EGID, Lille, France; 7 Biostatistics and Bioinformatics, Milken Institute School of Public Health, George Washington University, Rockville, MD USA; 8 Biostatistics and Medical Informatics Research Group, Department of Public Health, Vrije Universiteit Brussel, Brussels, Belgium; 9 Nutrition, Eumetabolism and Health Group, Institut d’Investigació Biomèdica de Girona (IDIBGI-CERCA), Girona, Spain; 10 Department of Medical Sciences, University of Girona, Girona, Spain; 11 CIBER Fisiopatología de la Obesidad y Nutrición (CIBEROBN), Instituto de Salud Carlos III, Madrid, Spain; 12 Department of Diabetes, Endocrinology and Nutrition, Dr. Josep Trueta University Hospital, Girona, Spain; 13 Departments of Epidemiology, Erasmus MC University Medical Center, Rotterdam, The Netherlands; 14 Diabetic Complications Unit, Lund University Diabetes Centre, Department of Clinical Science, Lund University, Malmö, Sweden; 15 Translational Medicine and Clinical Pharmacology, Boehringer Ingelheim Pharma GmbH & Co. KG, Ingelheim am Rhein, Germany; 16 Center for Thrombosis and Haemostasis, University Medical Center of the Johannes Gutenberg-University Mainz, Mainz, Germany; 17 DZHK (German Center for Cardiovascular Research), Partner Site Rhine-Main, Mainz, Germany; 18 Institute of Molecular Biology (IMB), Mainz, Germany; 19 School for Cardiovascular Diseases, Maastricht University, Maastricht, The Netherlands; 20 Diabetes Complications Research Centre, Conway Institute, University College Dublin, Dublin, Ireland; 21 Data Science and Biostatistics Department, Julius Global Health, University Medical Center Utrecht, Utrecht, The Netherlands; 22 Department of Epidemiology, Harvard T.H. Chan School of Public Health, Boston, MA USA; 23 Genetics of Complex Traits, College of Medicine and Health, University of Exeter, Exeter, UK; 24 Population Health and Genomics, University of Dundee, Dundee, UK

**Keywords:** Obesity, Diagnostic markers

Correction to: *Nature Medicine* 10.1038/s41591-024-03299-7, published online 24 October 2024.

In the version of the article initially published, Pierre Bauvin’s surname appeared incorrectly (as Bauvain) and has now been amended in the HTML and PDF versions of the article.

