# Supplementary material for: Subclassification of obesity for precision prediction of cardiometabolic diseases
Source: Nat Med. 2024 Oct 24;31(2):534–43. doi: 10.1038/s41591-024-03299-7 (PMC11835733; doi:10.1038/s41591-024-03299-7)
Supplement: Supplementary file 1 — Supplementary Figs. 1–26. [file 41591_2024_3299_MOESM1_ESM.pdf]

nature medicine

Supplementary information https://doi.org/10.1038/s41591-024-03299-7

Subclassification of obesity for precision prediction of cardiometabolic diseases

In the format provided by the authors and unedited

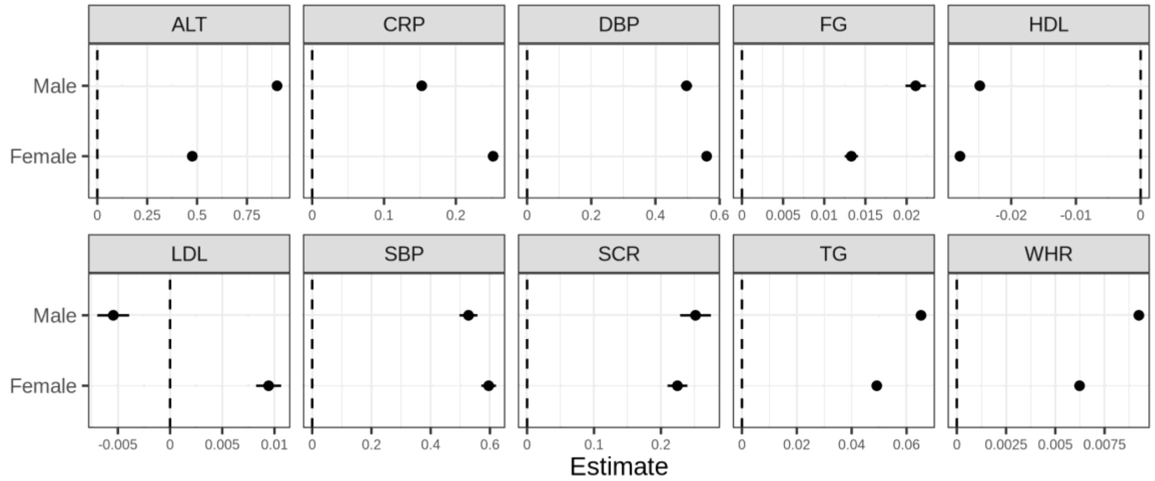

Supplementary Figure 1. Effect estimates of BMI in UKB. Age and current smoking adjusted effect estimates and 95% confidence intervals of BMI on biomarkers in UKB. Female sample size = 77,207. Male sample sizes = 67,904. UKB: UK Biobank. WHR: waist-to-hip ratio. SBP: systolic blood pressure. DBP: diastolic blood pressure. ALT: alanine transaminase. SCR: serum creatinine. CRP: C-reactive protein. HDL: high-density lipoprotein. TG: triglycerides. LDL: low-density lipoprotein. FG: fasting glucose.

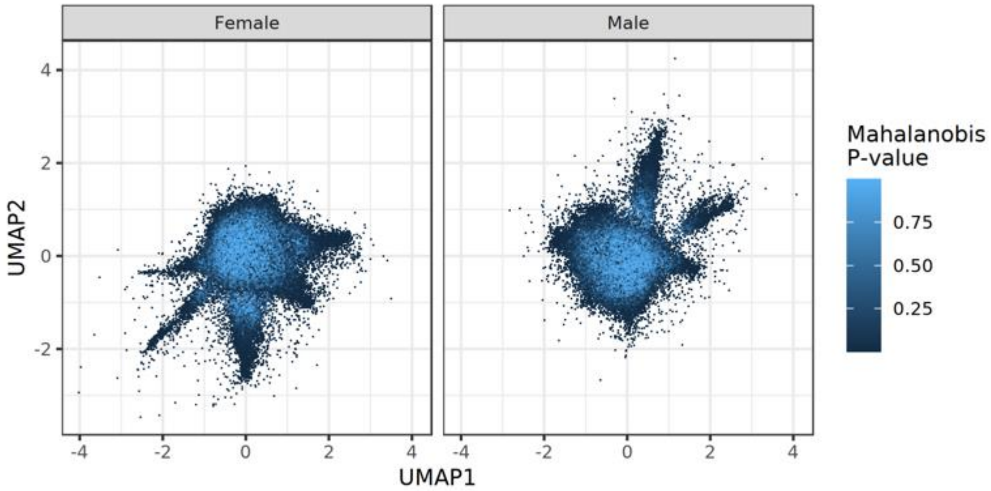

Supplementary Figure 2. UMAP projection of discordance in UKB. UMAP projection and distance from a multivariate normal distribution of residuals derived the BMI model, as measured by the Mahalanobis distance, converted to p-values. Lower p-values for an individual mean that it is less likely that their biomarker residuals come from a multivariate normal distribution centered around 0. P-values are derived from a Chi-square distribution with degrees of freedom equal to the number of variables included.

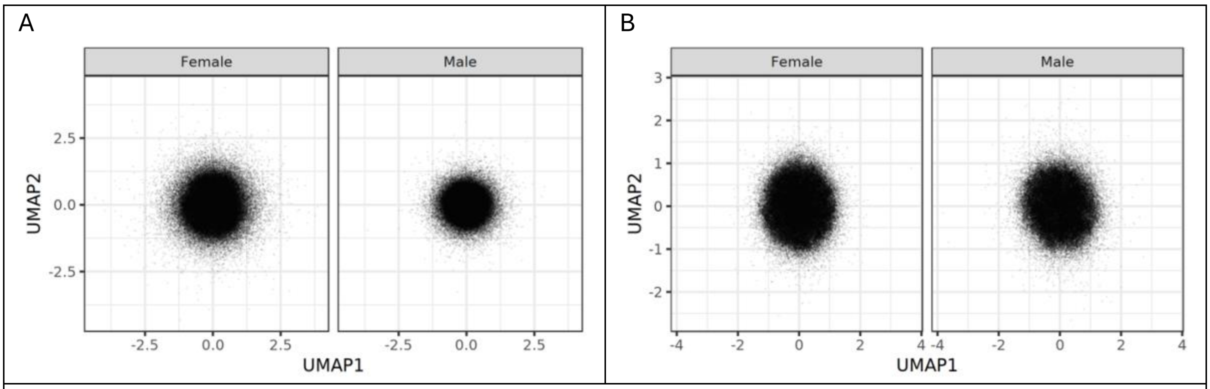

Supplementary Figure 3. Random UMAP projections of discordance data. UMAP projections of randomly generated multivariate normal distribution of residuals of the same size as the UK Biobank dataset used in the analysis. A: Assuming no correlation between biomarker residuals. B. With the same covariance structure as the residuals obtained in UK Biobank.

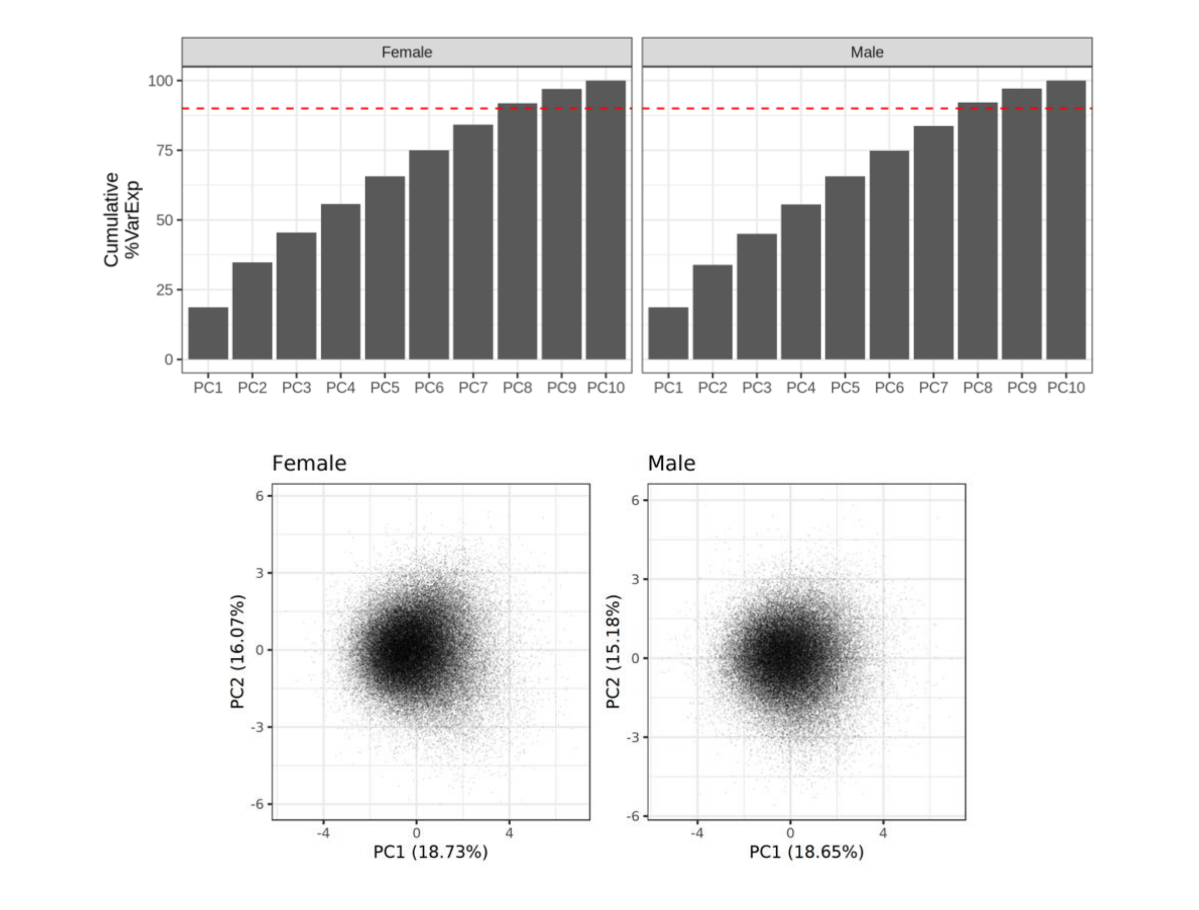

Supplementary Figure 4. Linear dimension reduction of discordance data. Principal component reduction of deviations from biomarkers from BMI-based expectations using principal component analysis. On top, cumulative variance explained by principal components. Bottom, scatter plot of PC 1 vs PC 2.

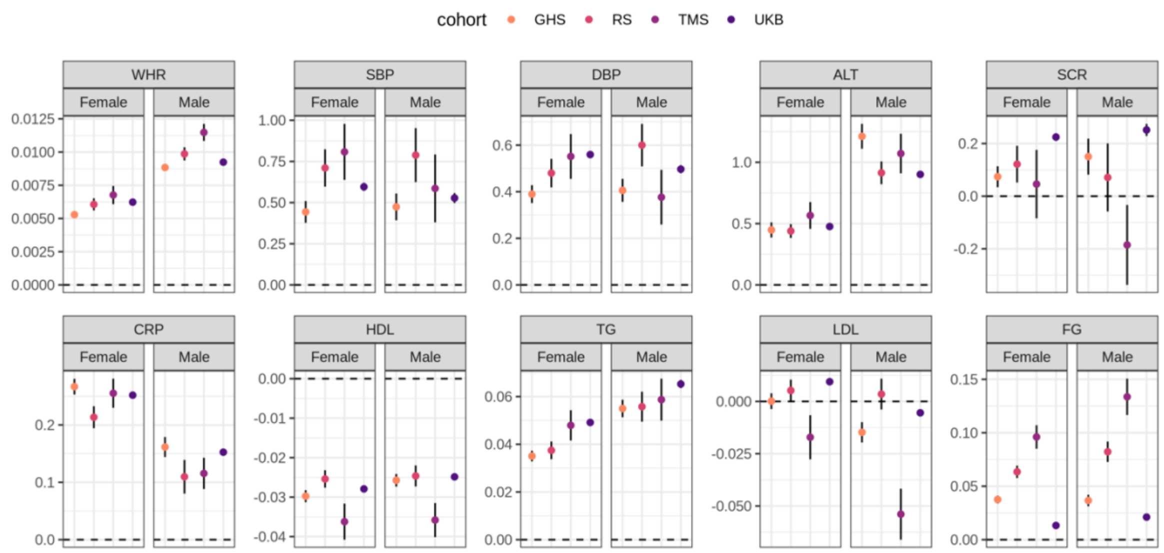

Supplementary Figure 5. Effect estimates of BMI. Age and current smoking adjusted effect estimates and 95% confidence intervals of BMI on biomarkers across cohorts. Female sample sizes: UKB = 77,207; TMS = 1,542; RS = 5,704; GHS = 7,301. Male sample sizes: UKB = 67,904; TMS = 1,633; RS = 4,289; GHS = 7,353. GHS: Gutenberg Health Study, TMS: Maastricht Study, RS: Rotterdam Study, UKB: UK Biobank. WHR: waist-to-hip ratio. SBP: systolic blood pressure. DBP: diastolic blood pressure. ALT: alanine transaminase. SCR: serum creatinine. CRP: C-reactive protein. HDL: high-density lipoprotein. TG: triglycerides. LDL: low-density lipoprotein. FG: fasting glucose.

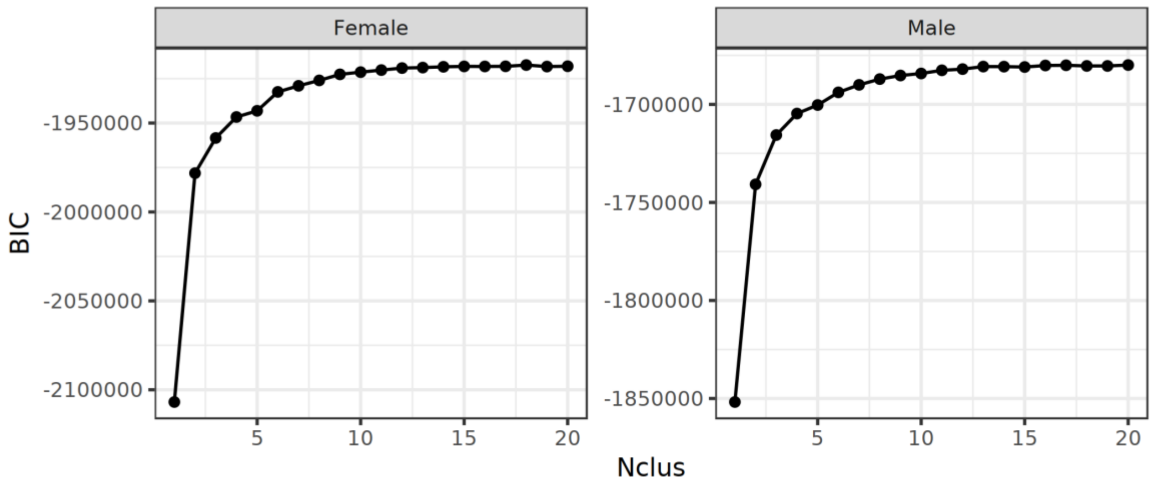

Supplementary Figure 6. Best partition of centroid-based clustering. Finding the best partition of a Gaussian mixture model applied on the deviation data. Each point is the Bayesian information criterion (BIC) from a model with the corresponding number of clusters. We considered an elbow at 6 clusters for both sexes, after which the BIC improvement flattens.

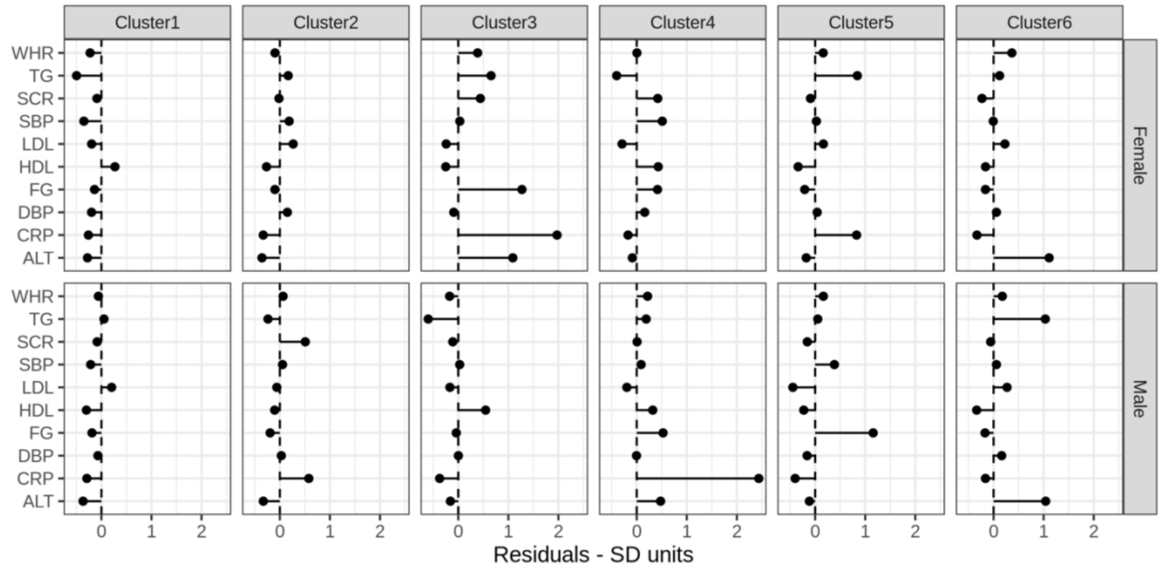

Supplementary Figure 7. Characteristics of centroid-based clusters. Cluster centres of best partitions from the Gaussian mixture modelling. Points represent average residual values within each profile, and the lines depict the distance from the null residual value. WHR: waist-to-hip ratio. TG: triglycerides. SCR: serum creatinine. SBP: systolic blood pressure. LDL: low-density lipoprotein. HDL: high-density lipoprotein. FG: fasting glucose. DBP: diastolic blood pressure. CRP: C-reactive protein. ALT: alanine transaminase.

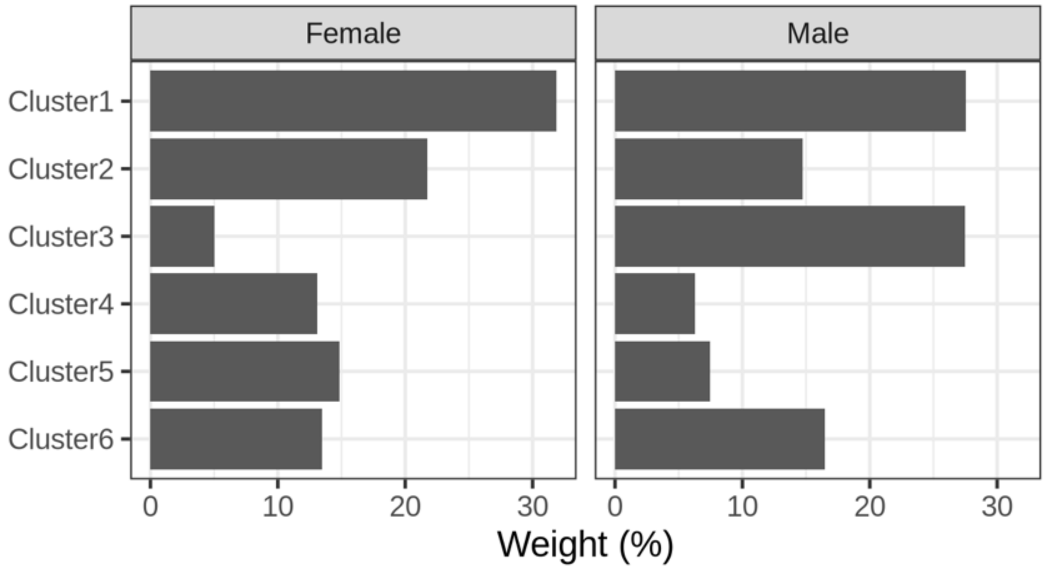

Supplementary Figure 8. Weights of centroid-based clusters. Cluster weights of best partitions from the Gaussian mixture modelling.

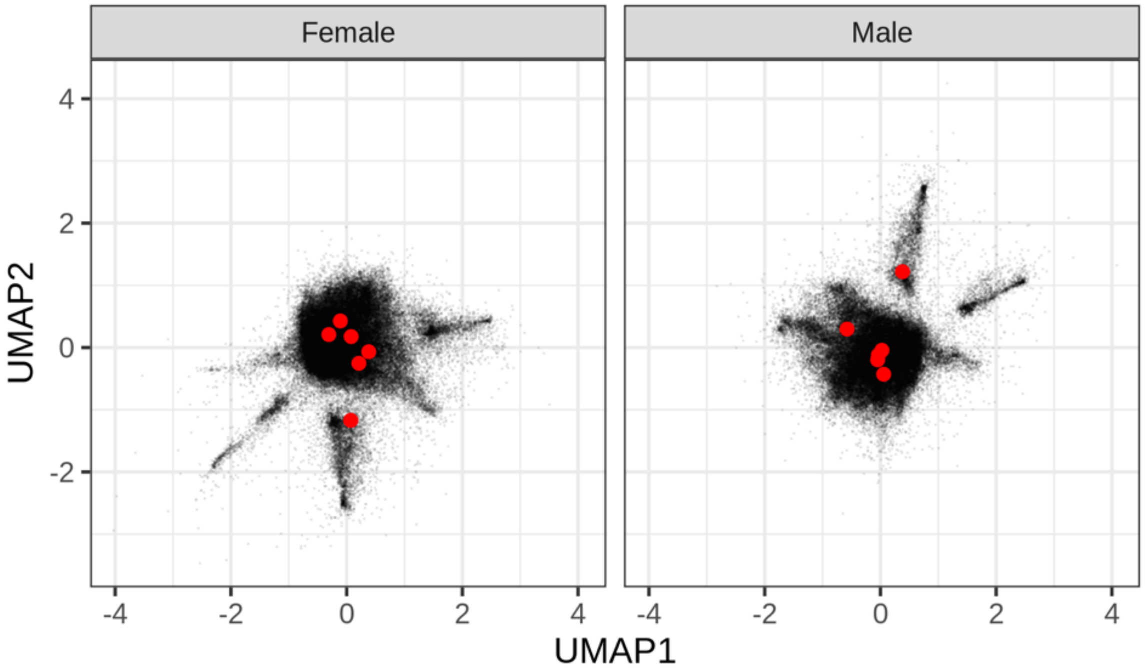

Supplementary Figure 9. Centroid-based results in UMAP of discordance. Overlaying cluster centres of best partitions from the Gaussian mixture modelling onto the UMAP projection

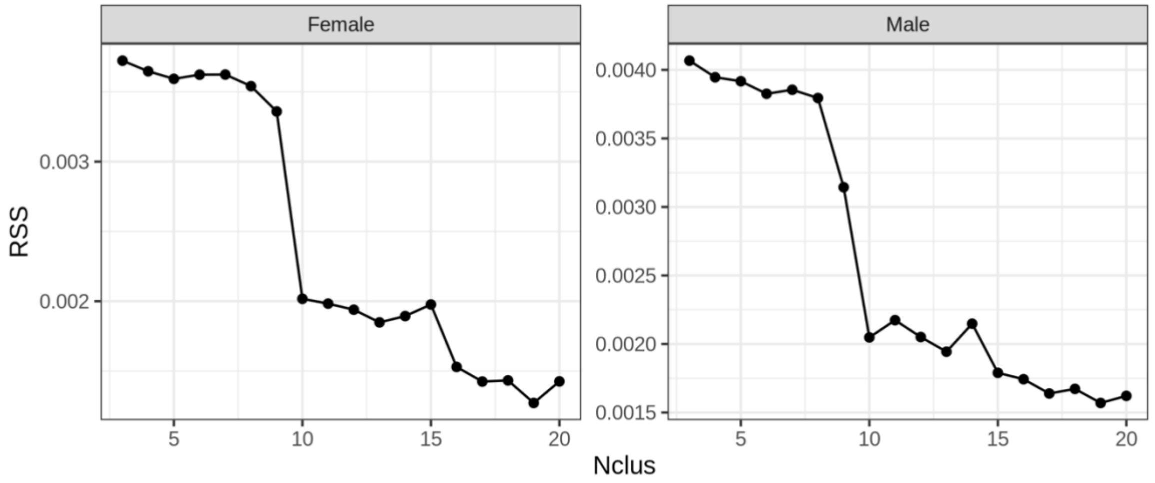

Supplementary Figure 10. Best partition of boundary-based clustering. Finding the best partition of an archetypal model applied on the deviation data. We considered an elbow at 11 clusters for both sexes, after which the residual sum of squares (RSS) change stalls.

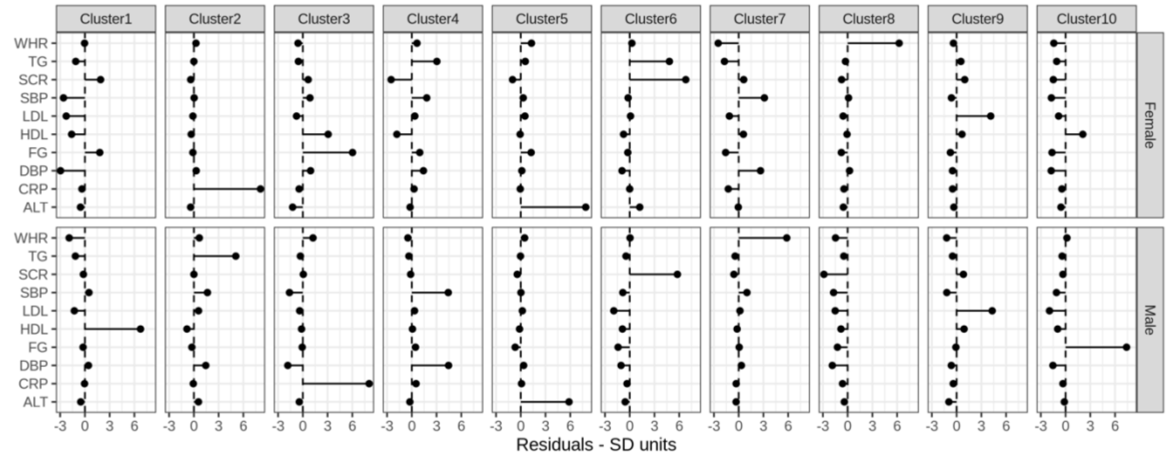

Supplementary Figure 11. Characteristics of boundary-based clusters. Centres of clusters from best partition using archetypal data. Points represent average residual values within each profile, and the lines depict the distance from the null residual value. WHR: waist-to-hip ratio. TG: triglycerides. SCR: serum creatinine. SBP: systolic blood pressure. LDL: low-density lipoprotein. HDL: high-density lipoprotein. FG: fasting glucose. DBP: diastolic blood pressure. CRP: C-reactive protein. ALT: alanine transaminase.

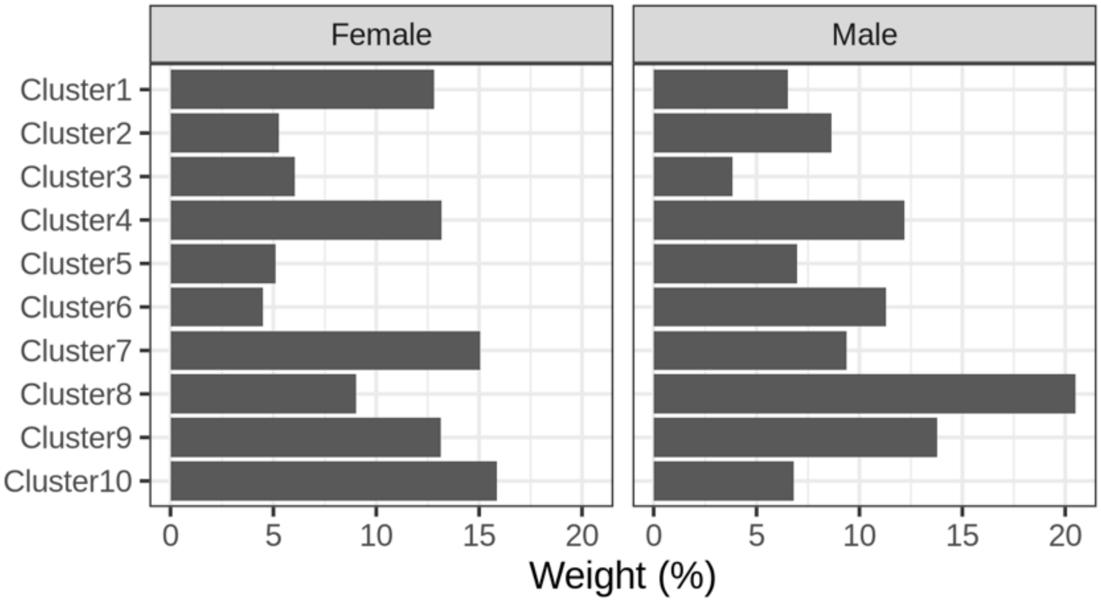

Supplementary Figure 12. Weights of boundary-based clusters. Cluster weights of best partitions from archetypal modelling.

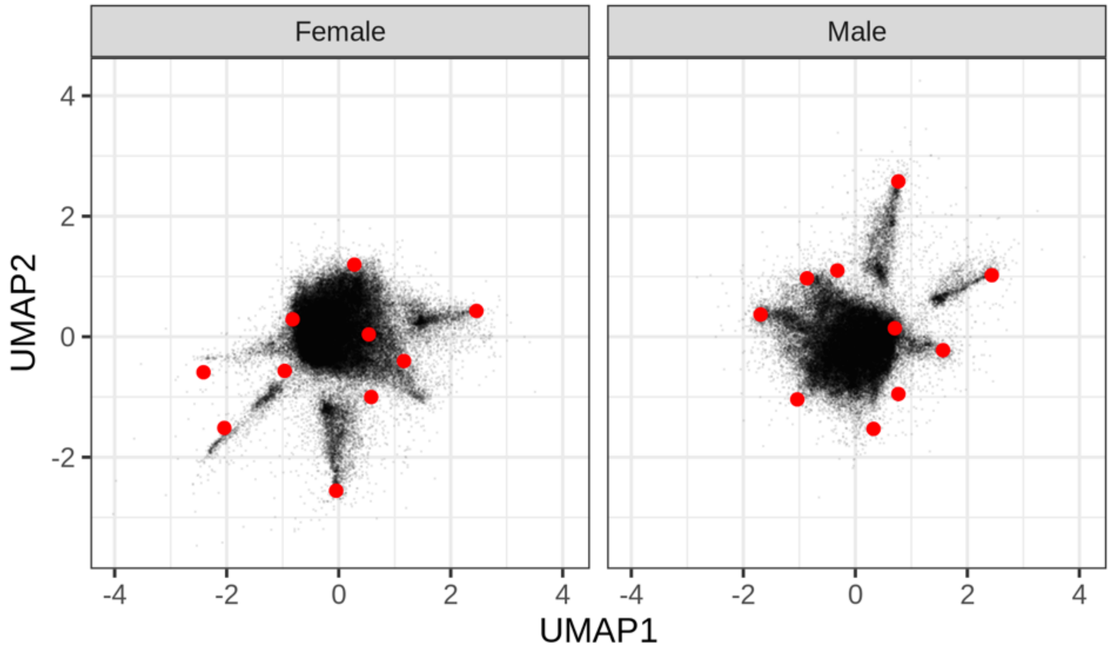

Supplementary Figure 13. Boundary-based results in UMAP of discordance. Overlaying cluster centres of best partitions from the archetypal modelling onto the UMAP projection.

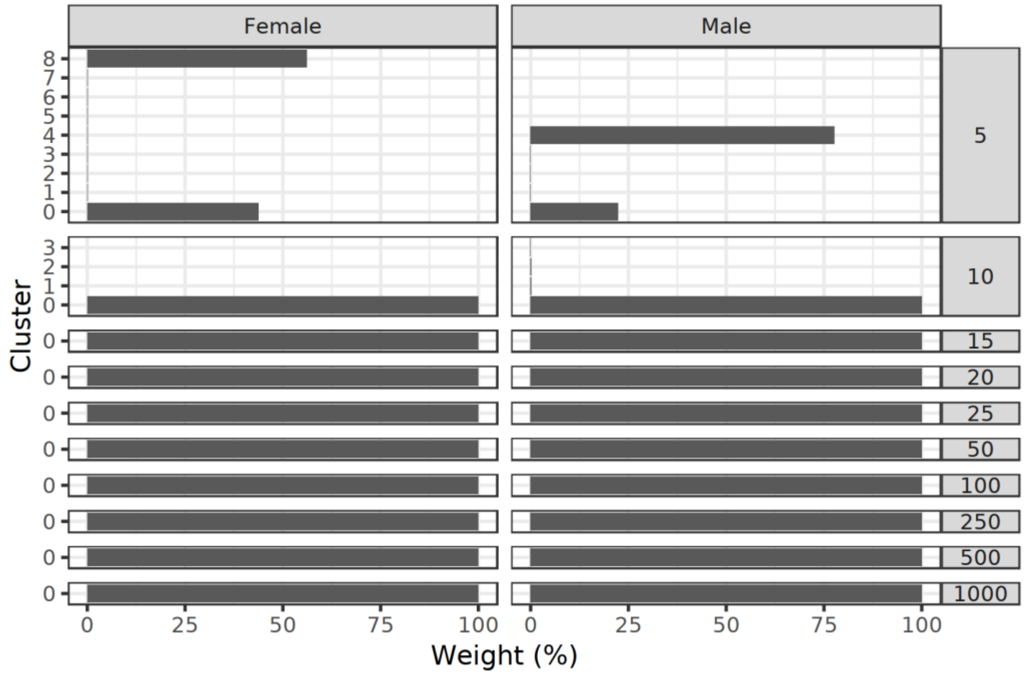

Supplementary Figure 14. Partitions of density-based clustering. Number of clusters and cluster weights for each run of HDBSCAN applied on discordance data using different values of the minPts argument.

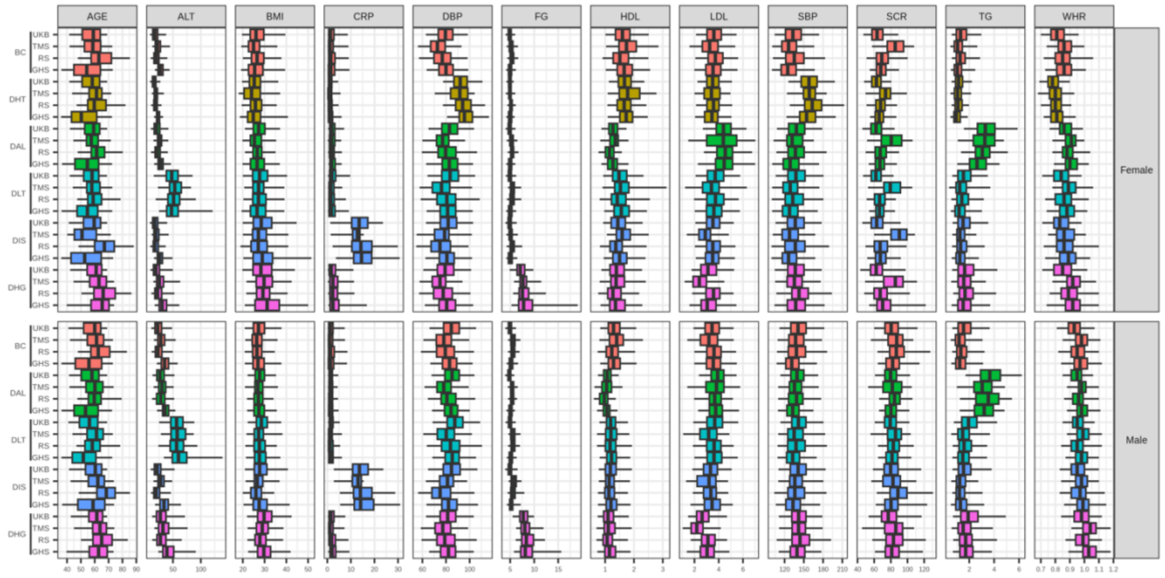

Supplementary Figure 15. Distribution of biomarker values per profile. Boxplots showing the distribution of continuous variables across cohorts and profiles. In each boxplot, the centre represents the median, the bounds of the box represent the interquartile range, and the whiskers represent the 2.5 and 97.5 percentiles. Female sample sizes: UKB = 77,207; TMS = 1,542; RS = 5,704; GHS = 7,301. Male sample sizes: UKB = 67,904; TMS = 1,633; RS = 4,289; GHS = 7,353. GHS: Gutenberg Health Study, TMS: Maastricht Study, RS: Rotterdam Study, UKB: UK Biobank. BC: Baseline concordant profile. DHT: discordant hypertensive profile. DAL: discordant adverse lipid profile. DLT: discordant liver transaminases profile. DIS: discordant inflammatory state profile. DHG: discordant hyperglycaemic profile. ALT: alanine transaminase. CRP: C-reactive protein. DBP: diastolic blood pressure. FG: fasting glucose. HDL: high-density lipoprotein. LDL: low-density lipoprotein. SBP: systolic blood pressure. SCR: serum creatinine. TG: triglycerides. WHR: waist-to-hip ratio.

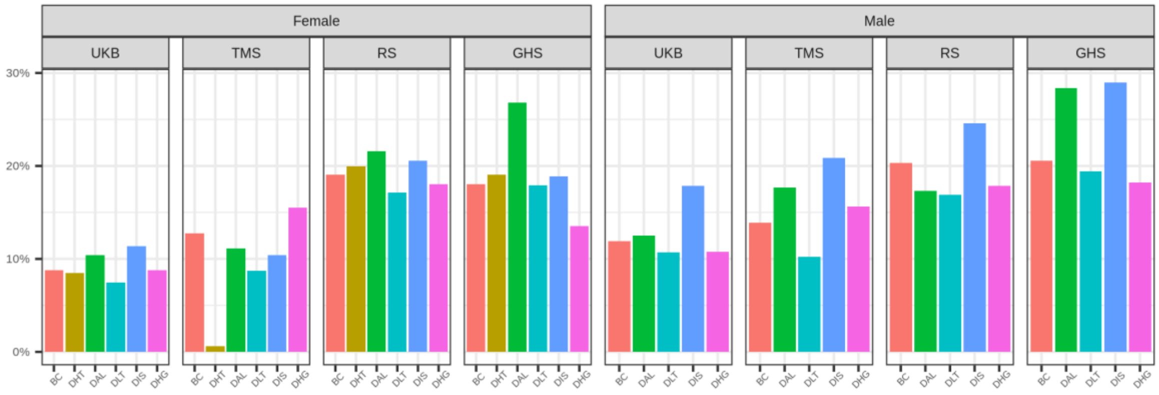

Supplementary Figure 16. Current smoking by profile. Proportion of current smokers per profile. GHS: Gutenberg Health Study, TMS: Maastricht Study, RS: Rotterdam Study, UKB: UK Biobank.

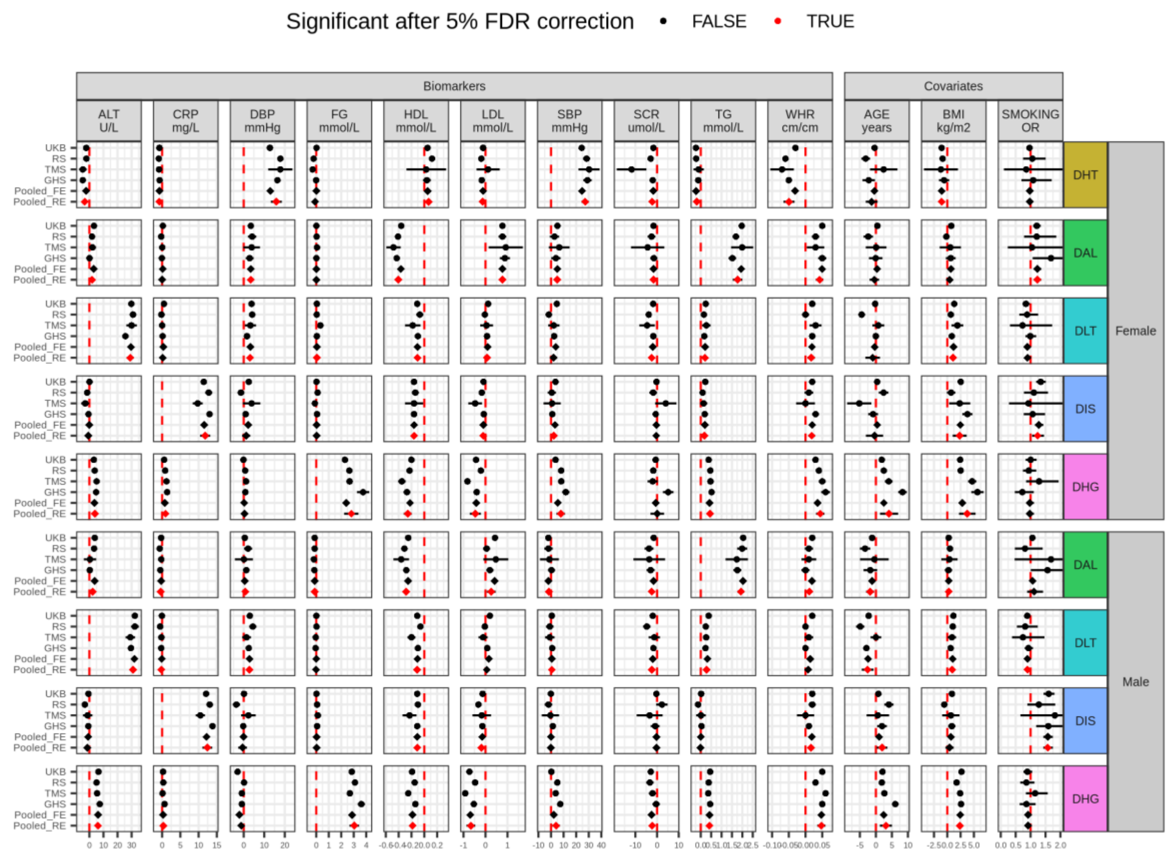

Supplementary Figure 17. Biomarker differences between the concordant and discordant profiles. Difference in absolute values of biomarkers and odds ratio of current smoking of discordant clusters compared to the concordant cluster. Lines represent 95% confidence intervals. Red estimates represent pooled estimates using a random effects meta-analysis that reached FDR 5% corrected statistical significance. Pooled female sample size = 91,754. Pooled male sample size = 81,178.

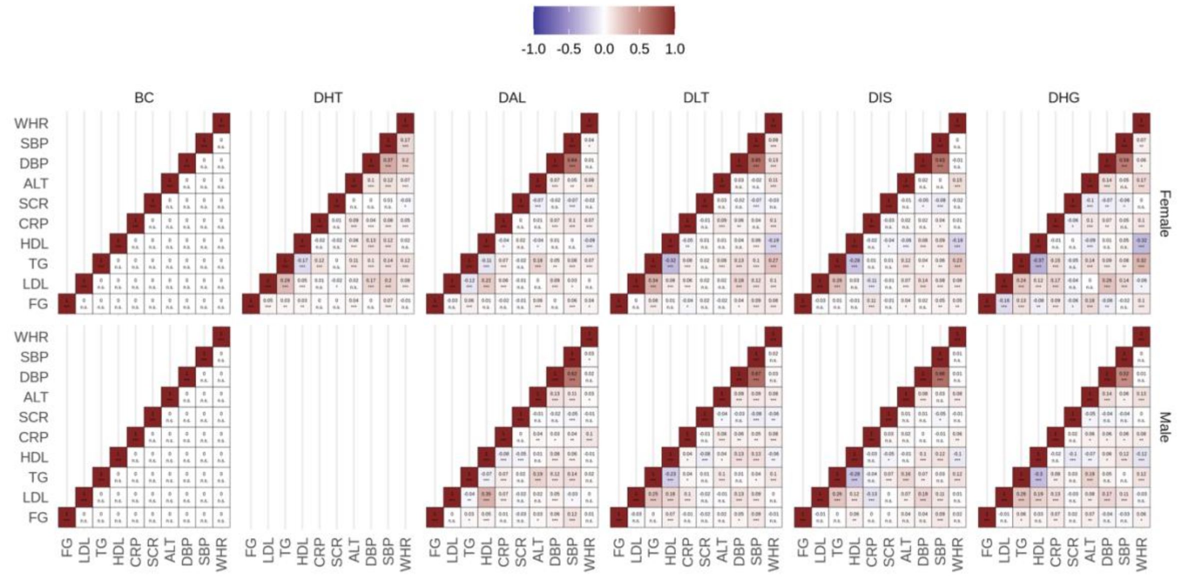

Supplementary Figure 18. Profile correlation matrices. Pearson correlation matrices of biomarker deviations from the expected for the BMI derived from the UK Biobank. Correlation p-values were derived from the t-distribution with degrees of freedom equal to the sample size minus 2 (Bessel's correction). Correlations and tests of significance are given in each cell. n.s., non-significant. * p-value < 0.05-. ** p-value < 0.01, *** p-value < 0.001. Female sample size = 77,207. Male sample size = 67,904. BC: Baseline concordant profile. DHT: discordant hypertensive profile. DAL: discordant adverse lipid profile. DLT: discordant liver transaminases profile. DIS: discordant inflammatory state profile. DHG: discordant hyperglycaemic profile. ALT: alanine transaminase. CRP: C-reactive protein. DBP: diastolic blood pressure. FG: fasting glucose. HDL: high-density lipoprotein. LDL: low-density lipoprotein. SBP: systolic blood pressure. SCR: serum creatinine. TG: triglycerides. WHR: waist-to-hip ratio.

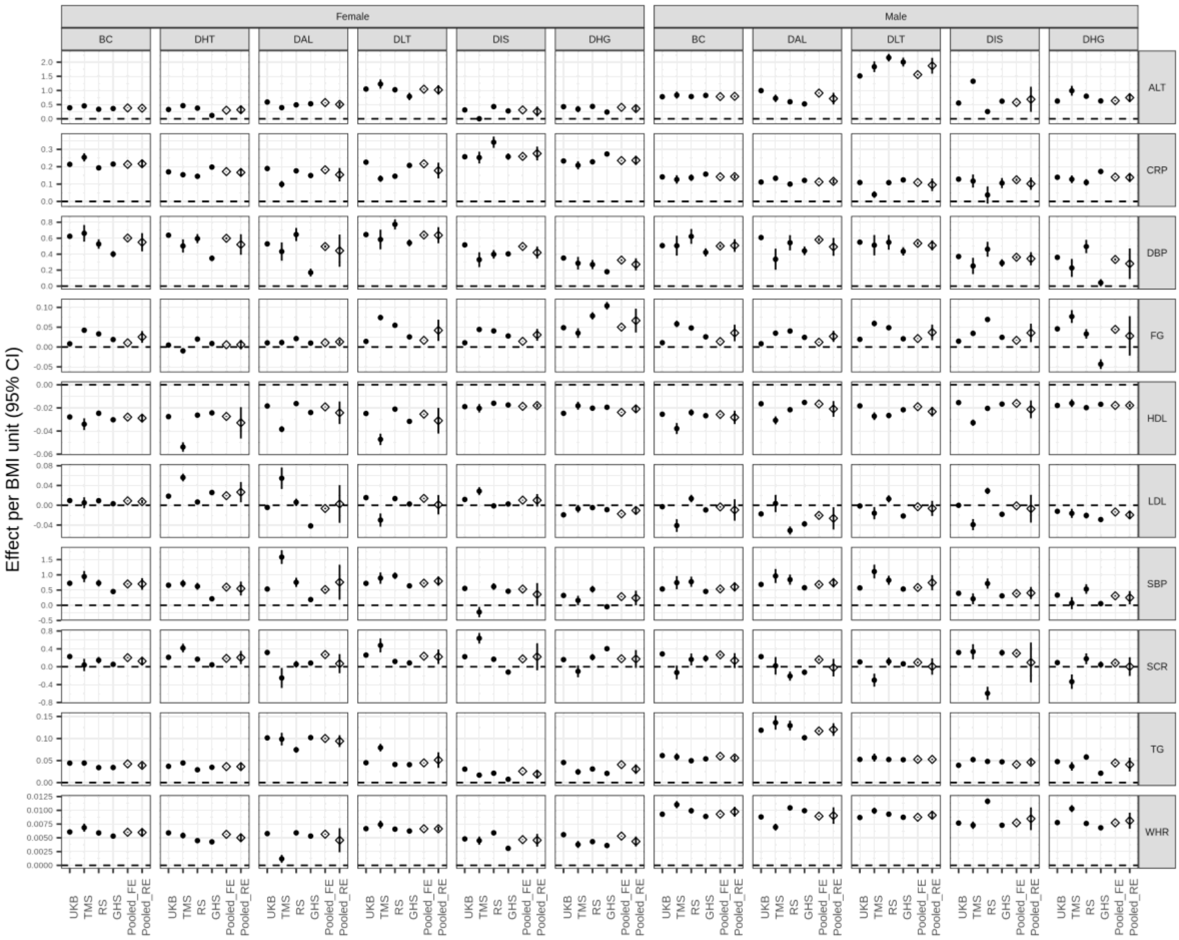

Supplementary Figure 19. Effect of BMI on biomarkers across profiles. Age and current smoking adjusted effect estimates and 95% confidence intervals of BMI on biomarkers across cohorts. Diamonds represent pooled estimates from random effects meta-analysis. Pooled female sample size = 91,754. Pooled male sample size = 81,178. GHS: Gutenberg Health Study, TMS: Maastricht Study, RS: Rotterdam Study, UKB: UK Biobank. BC: Baseline concordant profile. DHT: discordant hypertensive profile. DAL: discordant adverse lipid profile. DLT: discordant liver transaminases profile. DIS: discordant inflammatory state profile. DHG: discordant hyperglycaemic profile. ALT: alanine transaminase. CRP: C-reactive protein. DBP: diastolic blood pressure. FG: fasting glucose. HDL: high-density lipoprotein. LDL: low-density lipoprotein. SBP: systolic blood pressure. SCR: serum creatinine. TG: triglycerides. WHR: waist-to-hip ratio.

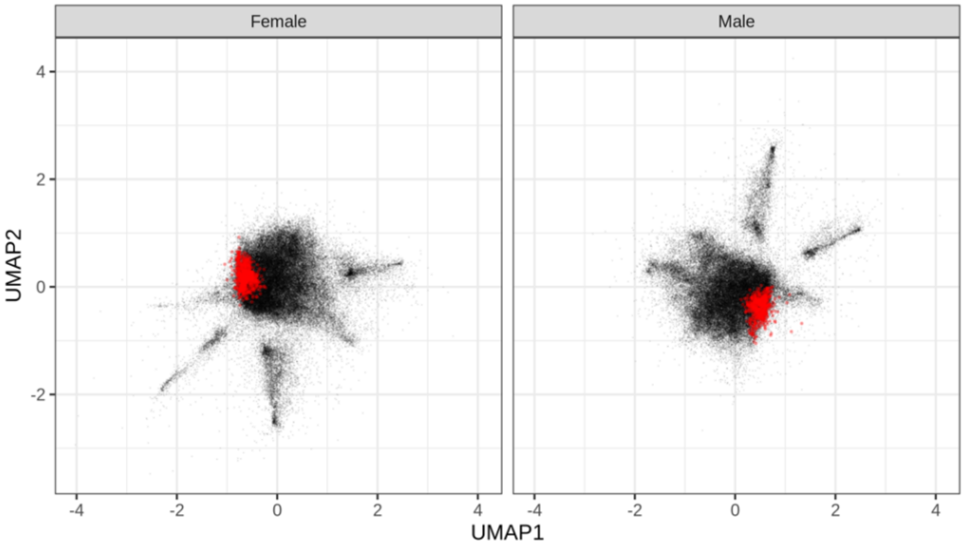

Supplementary Figure 20. Healthy discordant individuals in UMAP projection. Location of individuals with all biomarker values lower than expected for the BMI and higher than expected HDL for the BMI.

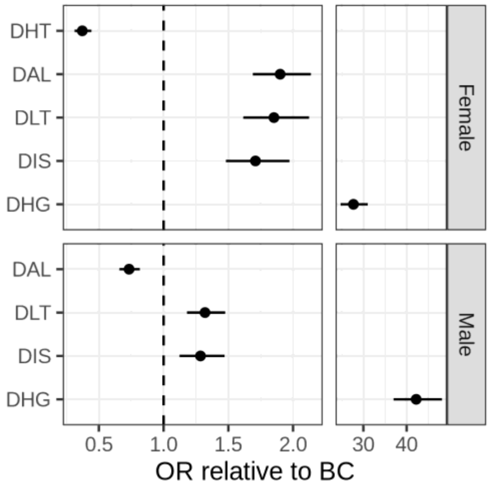

Supplementary Figure 21. Discordant profiles and the metabolic syndrome. Odds ratio and 95% confidence interval of the prevalence of metabolic syndrome in discordant profiles compared to the concordant profile in UKB. Female sample size = 77,207. Male sample size = 67,904. BC: Baseline concordant profile. DHT: discordant hypertensive profile. DAL: discordant adverse lipid profile. DLT: discordant liver transaminases profile. DIS: discordant inflammatory state profile. DHG: discordant hyperglycaemic profile. OR: odds ratio.

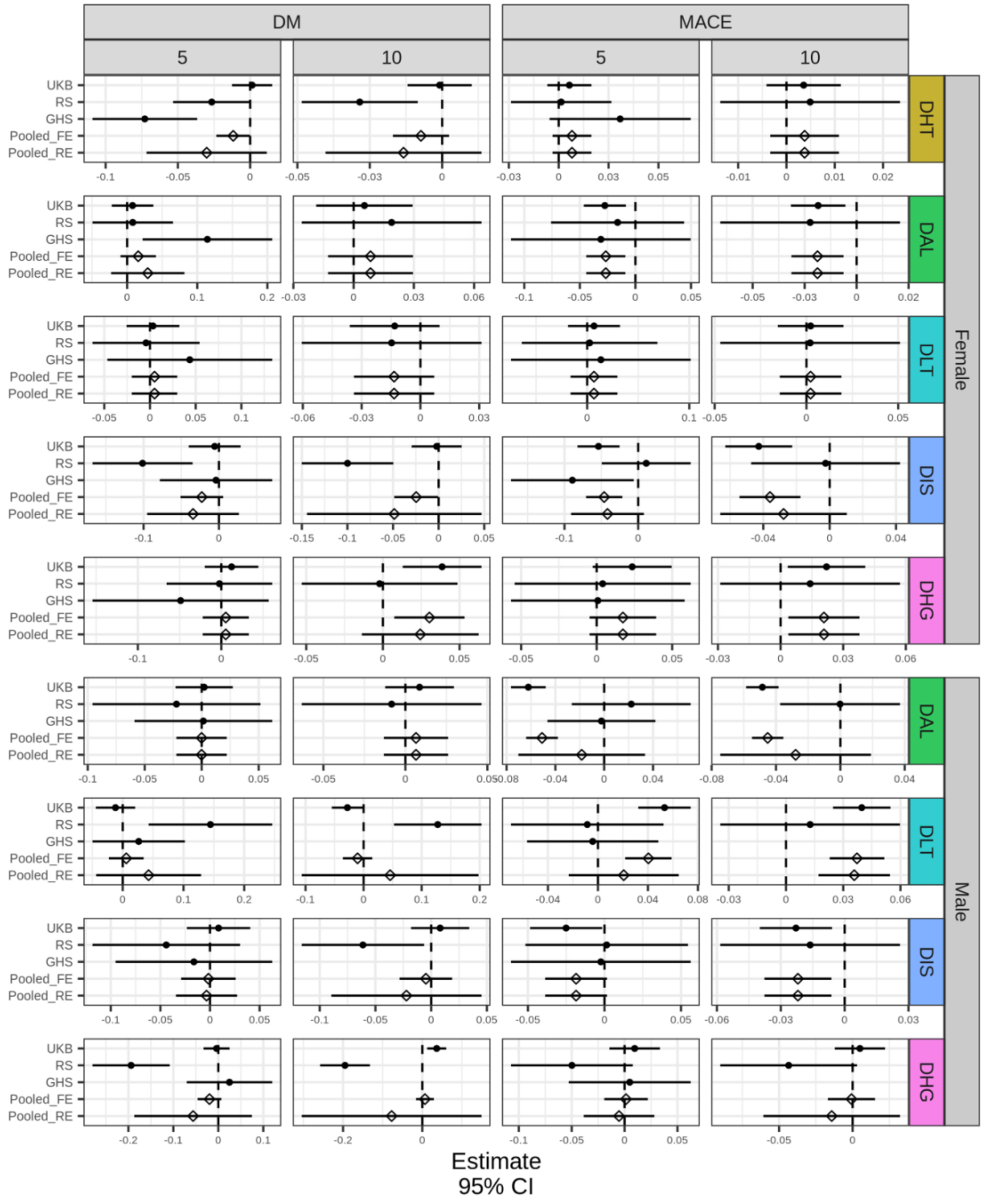

Supplementary Figure 22. Discordant profile log-ratio estimates. Study-specific and pooled discordant log-ratio estimates derived from the Cox regressions. Lines represent 95% confidence intervals. Female sample size for MACE = 85,392. Male sample size for MACE = 70,328. Female sample size for DM = 46,076. Male sample size for DM = 38,815. DM: Diabets Mellitus. MACE: Major Adverse Cardiovascular Event. UKB: UK Biobank, RS: Rotterdam Study, GHS: Gutenberg Health Study. BC: Baseline concordant profile. DLT: discordant liver transaminases profile. DIS: discordant inflammatory state profile. DHT: discordant hypertensive profile. DHG: discordant hyperglycaemic profile. DAL: discordant adverse lipid profile. Pooled_FE: pooled fixed effects. Pooled_RE: pooled random effects. BC: Baseline concordant profile. DHT: discordant hypertensive profile. DAL: discordant adverse lipid profile. DLT: discordant liver transaminases profile. DIS: discordant inflammatory state profile. DHG: discordant hyperglycaemic profile.

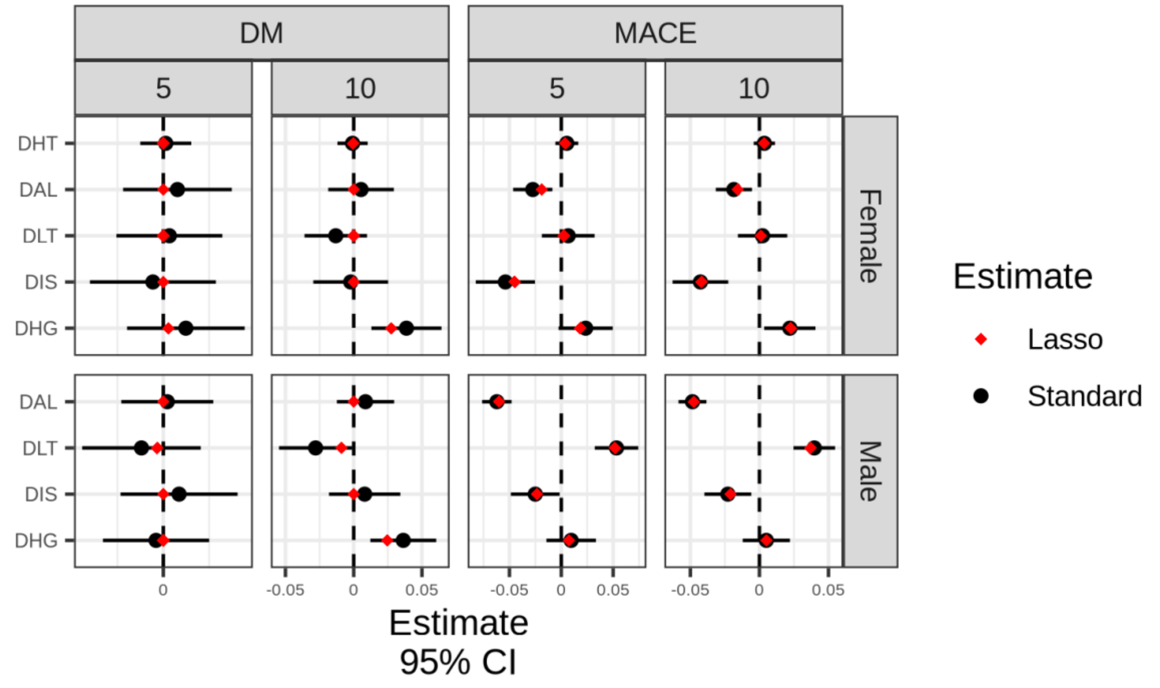

Supplementary Figure 23. Lasso regularisation of discordant estimates. Discordant log-ratio estimates derived from Cox regressions in the UKB were obtained using both standard models (as shown in Supplementary Figure 27) and regularized models with the Lasso penalty (red diamonds). The optimal penalty value was selected based on the lowest deviance in a 10-fold cross-validation procedure, performed using the 'glmnet' package in R. Lines represent 95% confidence intervals. Female sample sizes: MACE = 73,378; DM = 34,581. Male sample sizes: MACE = 60,348; DM = 29,006. UKB: UK Biobank. DM: Diabets Mellitus. MACE: Major Adverse Cardiovascular Event. BC: Baseline concordant profile. DHT: discordant hypertensive profile. DAL: discordant adverse lipid profile. DLT: discordant liver transaminases profile. DIS: discordant inflammatory state profile. DHG: discordant hyperglycaemic profile.

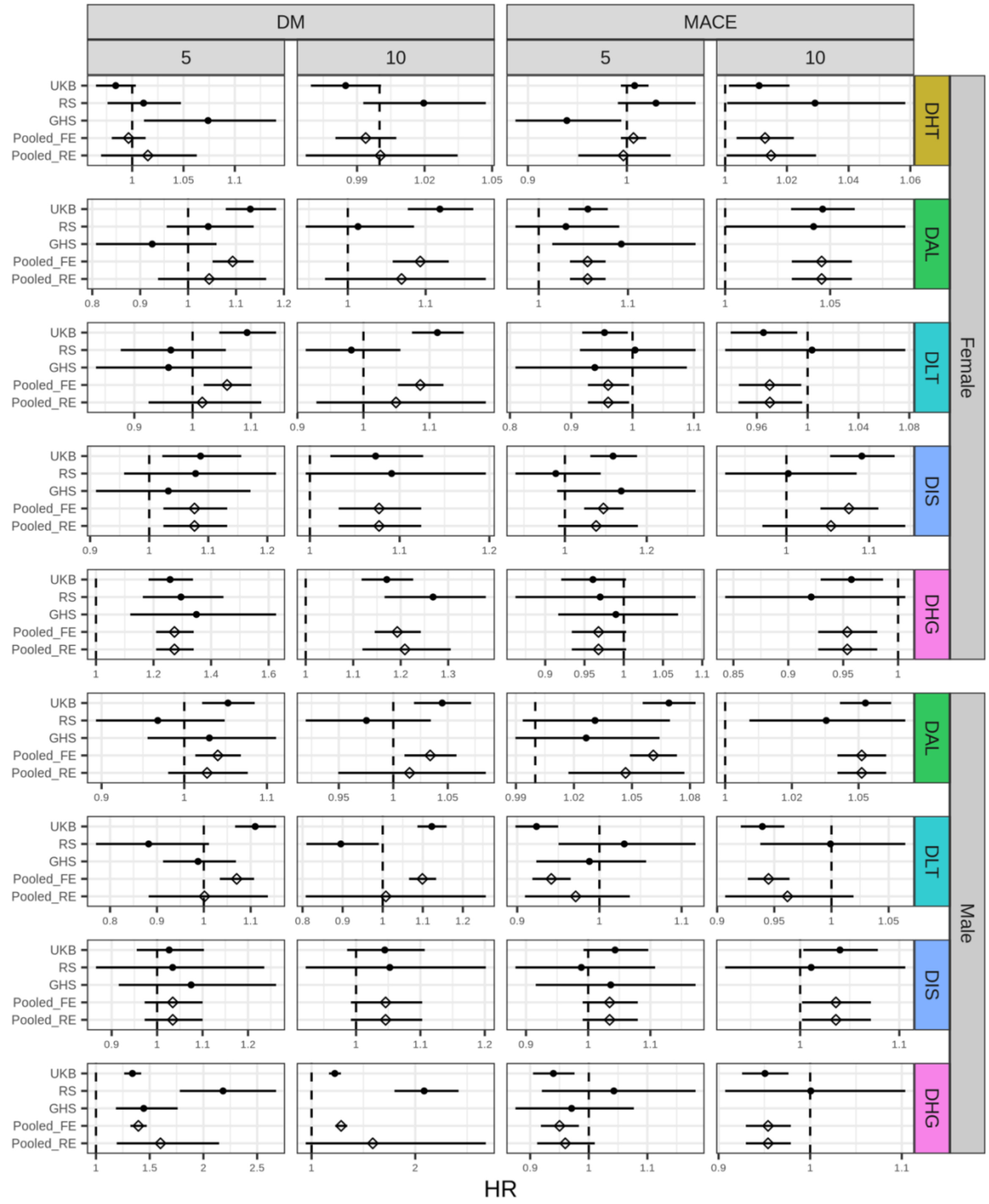

Supplementary Figure 24. Effect of shifting from concordant to a discordant profile. Study-specific and pooled estimates of a 10% shift from the concordant to each discordant profile. Lines represent 95% confidence intervals. Pooled female sample sizes: MACE = 85,392; DM = 46,076. Pooled male sample sizes: MACE = 70,328; DM = 38,815. UKB: UK Biobank, RS: Rotterdam Study, GHS: Gutenberg Health Study. DHT: discordant hypertensive profile. DAL: discordant adverse lipid profile. DLT: discordant liver transaminases profile. DIS: discordant inflammatory state profile. DHG: discordant hyperglycaemic profile. Pooled_FE: pooled fixed effects. Pooled_RE: pooled random effects.

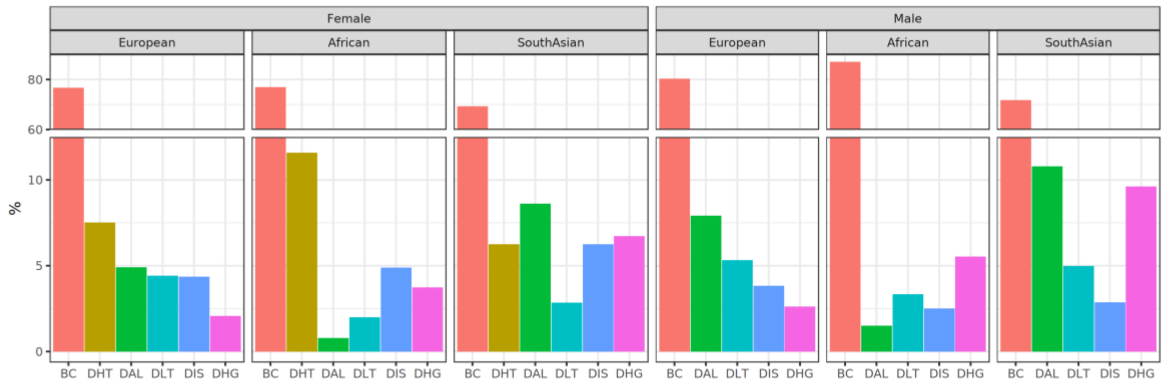

Supplementary Figure 25. Trans-ancestry profile proportions. Profile proportions in European, African and South Asian populations in UK Biobank. BC: Baseline concordant profile. DHT: discordant hypertensive profile. DAL: discordant adverse lipid profile. DLT: discordant liver transaminases profile. DIS: discordant inflammatory state profile. DHG: discordant hyperglycaemic profile.

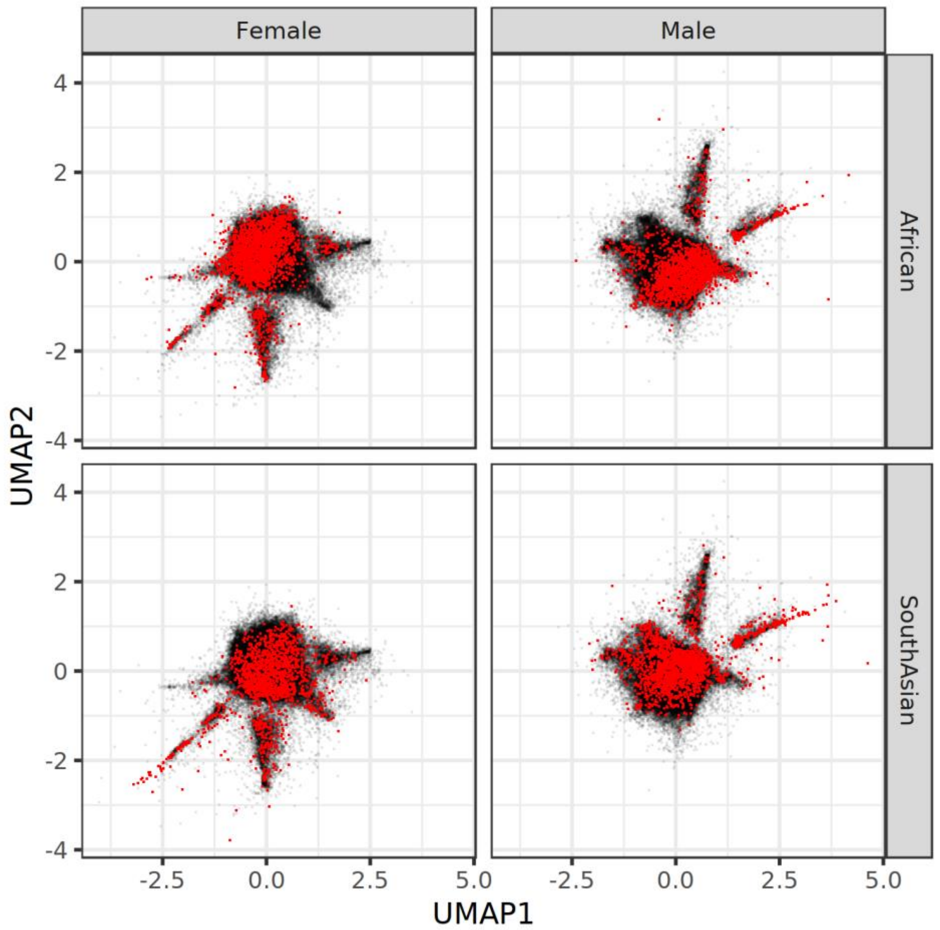

Supplementary Figure 26. Trans-ancestry UMAP overlay. Projection of African and South Asian populations from the UK Biobank on the European BMI-discordance projection.
