# Supplementary material for: Subclassification of obesity for precision prediction of cardiometabolic diseases
Source: Nat Med. 2024 Oct 24;31(2):534–43. doi: 10.1038/s41591-024-03299-7 (PMC11835733; doi:10.1038/s41591-024-03299-7)
Supplement: Supplementary file 2 — Reporting Summary [file 41591_2024_3299_MOESM2_ESM.pdf]

nature portfolio

Corresponding author(s): Paul W. Franks, Daniel E. Coral, Femke Smit

Last updated by author(s): Aug 20, 2024

Reporting Summary

Nature Portfolio wishes to improve the reproducibility of the work that we publish. This form provides structure for consistency and transparency in reporting. For further information on Nature Portfolio policies, see our Editorial Policies and the Editorial Policy Checklist.

Statistics

For all statistical analyses, confirm that the following items are present in the figure legend, table legend, main text, or Methods section.

n/a	Confirmed	
☐	☒	The exact sample size (*n*) for each experimental group/condition, given as a discrete number and unit of measurement
☐	☒	A statement on whether measurements were taken from distinct samples or whether the same sample was measured repeatedly
☐	☒	The statistical test(s) used AND whether they are one- or two-sided<br>*Only common tests should be described solely by name; describe more complex techniques in the Methods section.*
☐	☒	A description of all covariates tested
☐	☒	A description of any assumptions or corrections, such as tests of normality and adjustment for multiple comparisons
☐	☒	A full description of the statistical parameters including central tendency (e.g. means) or other basic estimates (e.g. regression coefficient) AND variation (e.g. standard deviation) or associated estimates of uncertainty (e.g. confidence intervals)
☐	☒	For null hypothesis testing, the test statistic (e.g. *F*, *t*, *r*) with confidence intervals, effect sizes, degrees of freedom and *P* value noted<br>*Give P values as exact values whenever suitable.*
☒	☐	For Bayesian analysis, information on the choice of priors and Markov chain Monte Carlo settings
☒	☐	For hierarchical and complex designs, identification of the appropriate level for tests and full reporting of outcomes
☐	☒	Estimates of effect sizes (e.g. Cohen's *d*, Pearson's *r*), indicating how they were calculated

Our web collection on statistics for biologists contains articles on many of the points above.

Software and code

Policy information about availability of computer code

Data collection	Data collected from all four cohorts were in tabular format and were analyzed using the R statistical software version 4.2.
Data analysis	All analyses were conducted in R version 4.2. To run UMAP, which is central in our analysis, we used the package uwot version 0.1.16. For graph-based clustering algorithms we used the implementations from the igraph package version 2.0.2. We provided all codes used in our analysis in a Github repository: https://github.com/danielcoral/SOPHIA_Cross_Sectional.

For manuscripts utilizing custom algorithms or software that are central to the research but not yet described in published literature, software must be made available to editors and reviewers. We strongly encourage code deposition in a community repository (e.g. GitHub). See the Nature Portfolio guidelines for submitting code & software for further information.

Data

Policy information about availability of data

All manuscripts must include a data availability statement. This statement should provide the following information, where applicable:

- Accession codes, unique identifiers, or web links for publicly available datasets
- A description of any restrictions on data availability
- For clinical datasets or third party data, please ensure that the statement adheres to our policy

UKB data are available through a procedure described at http://www.ukbiobank.ac.uk/using-the-resource/, where timeframe information can also be found. Restrictions apply to the availability of TMS data, which were used under license for the current study. Data are, however, available from the authors upon

reasonable request and with permission of TMS management team. Timelines and conditions can be found at https://www.demaastrichtstudie.nl/research/data-guidelines. Access to RS can be requested through the management team (secretariat.epi@erasmusmc.nl), which has a protocol for approving data requests. Because of restrictions based on privacy regulations and informed consent of the participants, data cannot be made freely available in a public repository. More information can be found at https://www.erasmusmc.nl/en/research/core-facilities/ergo-the-rotterdam-study. Data from GHS are not publicly available because this is not covered by the informed consent of participants. However, access to the data in the local database is possible upon reasonable request according to the ethics vote. Interested scientists can make their requests to the Gutenberg Health Study Steering Committee (e-mail: ed.zniam-shg@ofni). More information can be found at http://www.gutenberghealthstudy.org/.

Human research participants

Policy information about studies involving human research participants and Sex and Gender in Research.

Reporting on sex and gender	All our analyses were sex-stratified
Population characteristics	Female sample size: 91,754. Male sample size: 81,178. Average age: 58 years. A full description of the baseline characteristics of participants in all four studies is provided in Supplementary Tables 1 and 2.
Recruitment	In all four cohorts, invitations were sent to individuals residing in predefined areas. Because of this, healthy volunteers are more likely to participate. Noteworthy, the Maastricht study oversampled individuals with diabetes.
Ethics oversight	UKB was approved by the National Health Service's National Research Ethics Service (11/NW/0382). Analysis of individual-level data from UKB participants in Lund University was approved by the Swedish Ethical Review Authority (2021-0317). TMS has been approved by the institutional medical ethical committee (NL31329.068.10) and the Dutch Minister of Health, Welfare and Sports (131088-105234-PG). RS has been approved by the Medical Ethics Committee of Erasmus MC (MEC 02.1015) and by the Dutch Ministry of Health, Welfare, and Sport (1071272-159521-PG). The GHS was approved by the ethics committee of the Statutory Medical Board of Rhineland-Palatinate, Germany (reference no. 837.020.07). All studies conformed to the ethical principles for medical research involving human participants outlined in the Declaration of Helsinki. All participants provided written informed consent at enrolment.

Note that full information on the approval of the study protocol must also be provided in the manuscript.

Field-specific reporting

Please select the one below that is the best fit for your research. If you are not sure, read the appropriate sections before making your selection.

☒ Life sciences ☐ Behavioural & social sciences ☐ Ecological, evolutionary & environmental sciences

For a reference copy of the document with all sections, see nature.com/documents/nr-reporting-summary-flat.pdf

Life sciences study design

All studies must disclose on these points even when the disclosure is negative.

Sample size	No sample size calculation was performed. Our prior expectation is that for most individuals the relationship between BMI and biomarkers would be captured by a linear model, and that at least 5% of participants would heavily deviate from this expected relationship. In our discovery dataset, where the total number of participants exceeded 50,000 individuals per group, the expected number of outliers of the relationship between BMI and biomarkers would be over 2,500 individuals. We hypothesized that this latter group of individuals could be divided in at least 10 subgroups (the number of biomarkers we included in our analyses), which means that each group would have at least 250 individuals. This number is above the threshold that has been recommended previously to have enough statistical power for clustering analyses, for example, in Dalmaijer et al BMC Bioinformatics 2022.
Data exclusions	We excluded individuals whose BMI or biomarkers were 5 standard deviations away from the mean of each variable. We only used complete cases. In the Rotterdam cohort, we used data that had been previously imputed using multiple imputation algorithms (MICE).Given the predominantly European ancestry of all cohorts, and to prevent separating subgroups in our clustering analyses that were driven mainly by ancestry, we only included individuals of European ancestry in our analyses. We ran additional analysis separately for other ancestries in the UK Biobank.
Replication	To assess the validity of the partitions identified in UKB, we ran the same pipeline of network construction, 2-dimensional visualization and clustering in TMS, RS and GHS, with the parameters as described above, and compared their results to UKB. We assessed whether individuals allocated to a profile in the original model from the UKB with high certainty (i.e., a probability > 80%) also had a similar median probability of being allocated to a profile found in any of the other three 'validation' cohorts (again, with a probability > 80%). We considered a profile as having been replicated if this condition was met in all three validation cohorts, which ensured that only clusters represented in all three cohorts were included in the final model. We then readjusted the weights for each profile and focused all downstream analyses on these latter replicated clusters. The clusters were named according to the average residuals of all biomarkers.
Randomization	This study was conducted on population-based cohorts and therefore has not been randomized.
Blinding	This study was conducted on population-based cohorts and therefore has not been blinded.

Reporting for specific materials, systems and methods

We require information from authors about some types of materials, experimental systems and methods used in many studies. Here, indicate whether each material, system or method listed is relevant to your study. If you are not sure if a list item applies to your research, read the appropriate section before selecting a response.

Materials & experimental systems

n/a	Involved in the study
☒	☐ Antibodies
☒	☐ Eukaryotic cell lines
☒	☐ Palaeontology and archaeology
☒	☐ Animals and other organisms
☒	☐ Clinical data
☒	☐ Dual use research of concern

Methods

n/a	Involved in the study
☒	☐ ChIP-seq
☒	☐ Flow cytometry
☒	☐ MRI-based neuroimaging
